# Predictive factors for long-term survival in pancreatic ductal adenocarcinoma that underwent surgery: a systematic review and meta-analysis of literature

**DOI:** 10.1007/s13304-025-02382-z

**Published:** 2025-09-01

**Authors:** Vincenzo D’Ambra, Claudio Ricci, Carlo Ingaldi, Laura Alberici, Riccardo Casadei

**Affiliations:** 1 Division of Pancreatic Surgery, IRCCS, Azienda Ospedaliero Universitaria Di Bologna, Via Massarenti N.9, 40138 Bologna, Italy; 2 Department of Internal Medicine and Surgery (DIMEC), Alma Mater Studiorum, University of Bologna, S. Orsola-Malpighi Hospital, Via Massarenti N.9, 40138 Bologna, Italy; 3 Alma Mater Studiorum, University of Bologna, Via Massarenti N.9, 40138 Bologna, Italy

**Keywords:** Pancreatic cancer, PDAC, Pancreatectomy, Long-term survival, Predictive factors

## Abstract

Long-term survivors after pancreatic resection for PDAC are rare, constituting a specific subset of patients that remains poorly understood. This study aims to identify the clinic-pathological, molecular, and therapeutic factors for predicting long-term survival (LTS). A systematic review and random-effect meta-analysis were conducted. Inclusion criteria were PDAC histology, resected patients, studies reporting risk factors, and comparing two groups. The primary endpoint was to evaluate predictive factors for LTS in patients with PDAC who underwent surgery. Results were reported with the Mantel–Haenszel random effects model using Risk Ratio (RR) or Mean Difference (MD). Meta-regression analysis was used to clarify heterogeneity. Nineteen studies, involving a total of 5412 patients, were included: 1097 (20,3%) in group LTS and 4334 (79,7%) in group STS (short-term survivors). These factors were associated to LTS: small size (RR 1.53, 95% IC 1.14; 2.05); T1-T2 stage (RR 1.07, 95% IC 1.03; 1.11); N0 (RR 1.82, 95% IC 1.60; 2.09); AJCC Stage I (RR 2.28 95% IC 1.87; 2.79); low-grade G1-2 (RR 1.21, 95% IC 1.09; 1.34); R0 resection (RR 1.11, 95% IC 1.08; 1.13); low levels of CEA (MD − 4.41, 95% IC − 6.23; − 2.59) and Ca 19.9 (MD − 66.4, 95% IC: − 71.9; − 60.9); absence of perineural invasion (RR 0.93, 95% IC: 0.90; 0.96), lymph-vascular invasion (RR 0.87, 95% IC: 0.83; 0.91), venous invasion (RR 0.63, 95% IC: 0.48; 0.83) and perioperative transfusions (RR 0.56, 95% IC: 0.40; 0.79). Several factors are associated with an LTS. They can be considered reliable indicators for predicting tumor progression.

**Supplementary Information:**

The online version contains supplementary material available at 10.1007/s13304-025-02382-z.

## Introduction

Pancreatic Ductal Adenocarcinoma (PDAC) represents a significant challenge in the field of oncology, with a bleak prognosis and a projected increase in its ranking as the second leading cause of cancer-related mortality in the coming decade. Late diagnosis and the lack of effective non-surgical treatments contribute to the poor prognosis of this disease [1]. Despite the importance of early diagnosis and surgical intervention, only a mere 20% of PDAC patients qualify as eligible candidates for surgery [2]. Survival rates remain dishearteningly low; most patients affected by PDAC succumb within the first 2–3 years after surgical resection due to disease recurrence and metastatic spread, resulting in a 5-year actuarial survival of 20–25% and an effective survival rate of 10–20% [3].

Thus, long-term survivors (LTSs) after pancreatic resection for PDAC are rare, constituting a specific subset of patients that remains poorly understood. This lack of understanding is mainly due to the challenges in analyzing prognostic factors related to long-term survival. Pathological examination relies on tumor size, grade, margin status, and lymph node invasion to predict disease recurrence and prognosis. However, there are no conclusive results regarding their ability to predict prognosis [4].

The aim of this systematic review and meta-analysis of the current literature is to identify predictive factors for long-term survival in patients with PDAC that undergo pancreatic resection.

## Methods

### Search strategy

A systematic review was conducted according to the Cochrane Handbook recommendations [5]. The search strategy was based on PICO’s methodology [6]:

1. Population: patients with PDAC who underwent pancreatic resection.
2. Intervention: patients with a survival of at least 5 years (LTS group);
3. Control: patients with a survival of less than 5 years (STS group);
4. Outcomes: to evaluate predictive factors for long-term survival.

A systematic literature search was performed using the PubMed/Medline database. Non-English language or non-human studies were excluded. The search was conducted using the string: “(“PDAC” OR “Pancreatic Cancer” OR “pancreatic ductal adenocarcinoma” OR “pancreas cancer” OR “Pancreas adenocarcinoma”) AND (“long survival” OR “5 years survival” OR “10 year survival” OR “long survivors” OR “long term survival” OR “long surveillance” OR “long term surveillance” OR “long survivorship” OR “long term survivorship”) AND (“surgery” OR “pancreatectomy” OR “pancreaticoduodenectomy” OR “resection”)”.

The last research was performed on June 30, 2023. Studies were selected by reading titles and abstracts. In case of doubt, studies were selected by reading the full text to identify papers fulfilling the inclusion criteria. PRISMA flowchart was built [7]. This protocol has been registered with PROSPERO 2023 CRD42023472753.

### Inclusion, exclusion criteria, data collection process, items, and risk of bias assessment

The inclusion criteria were: (i) Pancreatic Ductal Adenocarcinoma histology; (ii) patients treated with pancreatic resection (iii) studies reporting prevalence and risk factors; (iv) studies comparing two groups; (v) articles written in English.

Exclusion criteria were: (i) special samples that do not represent the general population; (ii) studies with incomplete data, unclear data, or obvious errors; (iii) non-English-language; (iv) case report, meta-analyses, reviews, editorials, and expert opinions; (v) absence of the control group.

Long-term survival was defined as a patient alive at 5 years, regardless of recurrence. Studies with a different definition of long survival were excluded if data were not extractable. We chose the 5-year threshold as it is the most commonly used in the literature, allows for a larger sample size, and is the most widely accepted among experts, as confirmed by a recent survey of pancreatic surgeons [8].

After full-text reading of selected studies, all relevant data and various short were collected in an Excel spreadsheet. The following data were extracted from the literature: first author, publication year, study period, study area, study design, number of patients, sex, age, and risk factors. Two evaluators (VD and CR) independently screened the literature, extracted data, evaluated the quality, and cross-checked the data. Any disagreement was solved after a collegial discussion involving the first author (VD).

The quality assessment of the studies was carried out using the validated Methodological Index for Non-Randomized Studies (MINORS) [9].

Patients included in the study were divided into two groups: Long-Survivors (LTS group) and Short-Survivors (STS group). The clinic-pathological characteristics of these two groups were compared and analyzed. The endpoints of the study were to evaluate predictive factors for a long time survival in patients with PDAC that underwent pancreatic resection.

### Summary measurements and synthesis of the results

All parameters were reported as frequencies with percentages or mean and standard deviation (SD). The Mantel–Haenszel random effects model was used to calculate the effect sizes [10]. The results were reported as risk ratios (RRs) with 95% confidence intervals (95% CI) for discrete variables and as mean differences (MD) with 95% confidence intervals (95% CI) for continuous variables. A two-tailed *p* value < 0.05 indicated a non-negligible effect.

### Risk of bias across studies and meta-regression analysis

The risk of bias across included studies was tested, measuring the “between-study heterogeneity” and publication bias. The heterogeneity between studies was tested using the I^2^ [11]. The heterogeneity was interpreted as follows: If *I*^2^ was < 50%, the risk of “between-study” heterogeneity was considered low–moderate, and if *I*^2^ was ≥ 50%, it was judged high. The meta-regression analysis was carried out if the heterogeneity was high and the result was statistically relevant [12]. In the first step, we calculated the distribution of confounding covariates among each arm, reporting the results as Risk Ratio (RR), Mean Difference (MD), or percentage. In the second step, *β*-coefficient with standard error (SE) and *R*^2^ was reported. The beta coefficient ± SE was related to the change in the RR or MD of the event: a positive beta coefficient means that the covariate increases the rate generating a positive HR modification. The *R*^2^ measured the quote, in percentage, of the heterogeneity explained by the variable. A two-tailed *p* value < 0.05 was judged significant. The *p* values were also recalculated using Monte Carlo permutation to obtain solid results. Publication bias was assessed with a funnel plot and Egger’s test, and a *p* value < 0.075 indicated a non-negligible “small-study effect” [13]. The trim-and-fill method was used to identify and correct potential publication bias [14].

This meta-analysis was performed using the statistical software Stata/SE (version 18.0).

## Results

### Search results and baseline characteristics

**Fig. 1:**
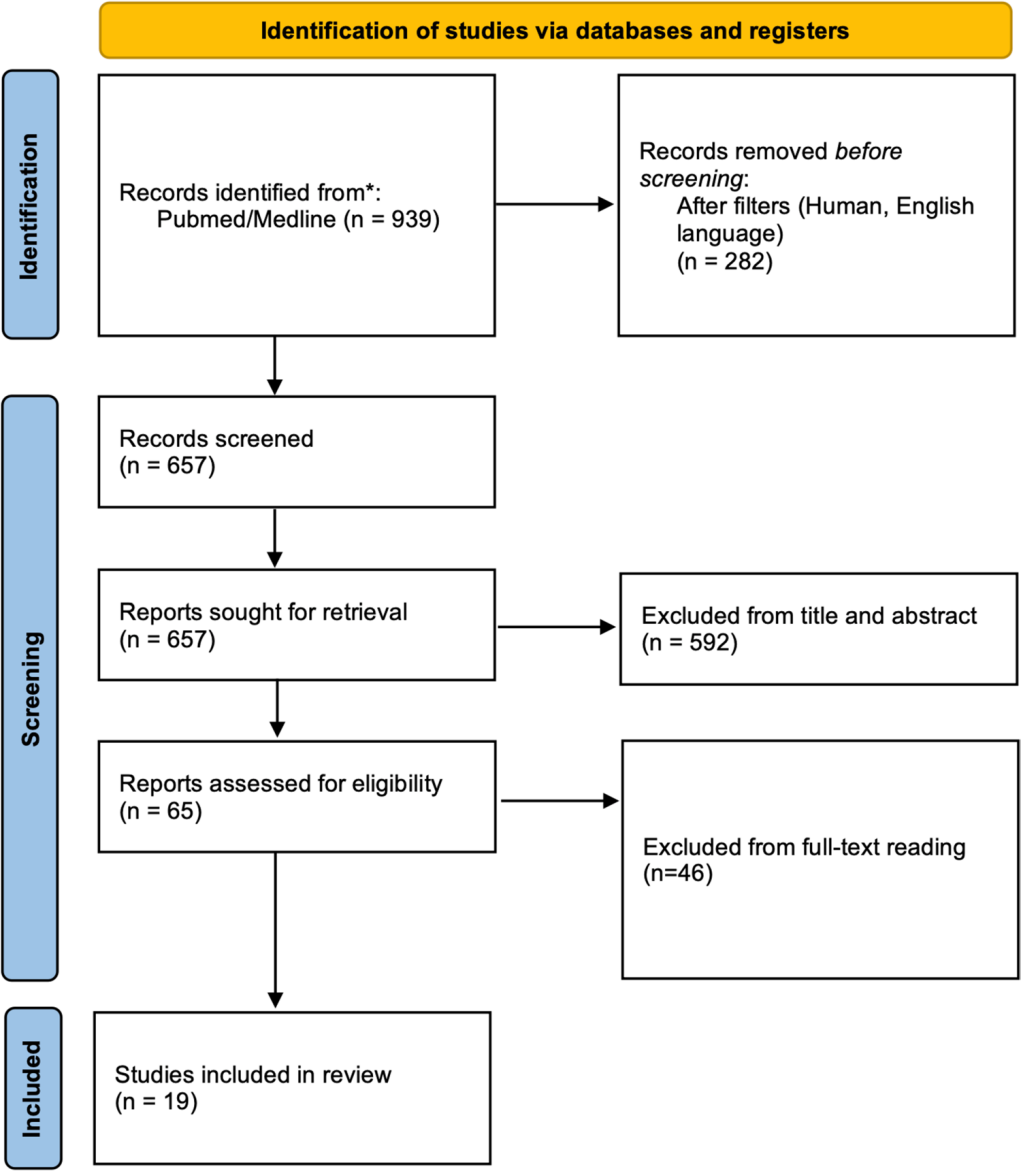
PRISMA Flow-chart of the study search and screening process

**Fig. 2:**
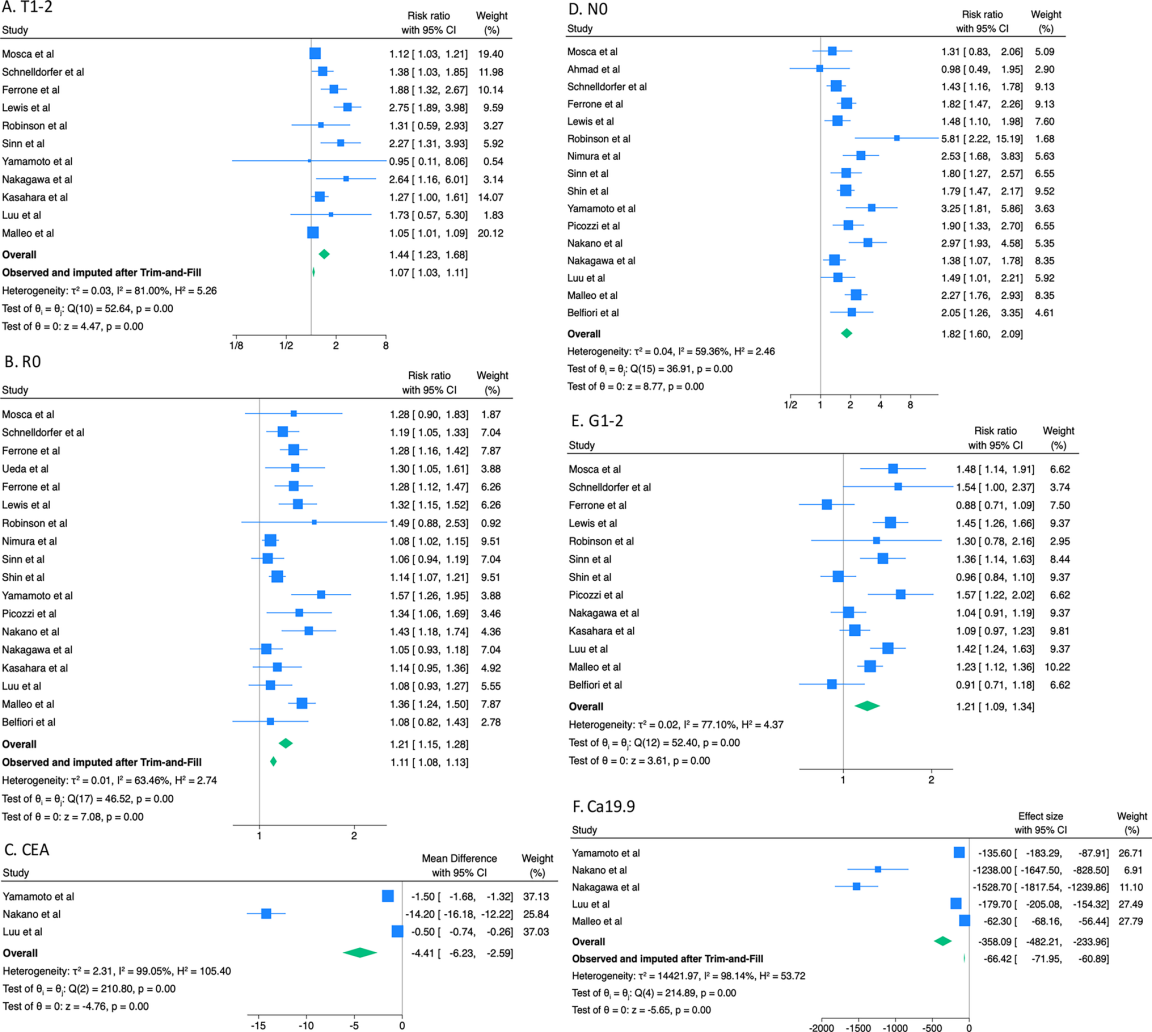
Main forest plots. Overall effect favors LTS if RR > 1 or MD > 0

The selection process is described in Fig. 1. The search identified a total of 939 records, 282 studies were excluded after filters because they were in non-English language or not conducted in humans. Of the remaining 657 papers, 592 were excluded because they were not pertinent to the field of study. Sixty-five articles were reviewed, and 46 of these were excluded. Finally, 19 studies [15–33] involving a total of 5412 patients were included: 1097 (20.3%) in group LTS and 4334 (79.7%) in group STS. Characteristics of the included studies are summarized in Table 1. There were 12 (63.2%) studies conducted in Western countries and 7 (36.8%) in Eastern. Seventeen were retrospective (89.5%), and only 2 (10.5%) were Randomized Controlled Trials. The median value of the MINORS score was 20 [18–21]. We identified 19 prognostic factors (Table 1). General characteristics of the population and all forest plots are described in Supplementary Material S-1. Table 2 describes an exhaustively meta-analysis of all outcomes. Main forest plots are depicted in Fig. 2. Meta-regression analysis is described in Supplementary Material S-2.

**Table 1:** Characteristics of included studies (n = 19)

Author	Year	Country	Study design	Time period	MINORS	No	LTS	STS	Risk factors evaluated
Mosca et al	1997	Italy	Retrospective	1980–1994	18	105	10	95	Sex, AJCC stage, R, Grading
Ahmad et al	2001	USA	Retrospective	1990–1998	19	125	24	101	Lymph-node involvement, Adj CT
Schnelldorfer et al	2008	USA	Retrospective	1981–2001	21	357	62	295	AJCC stage, R, LVI, PNI, Portal invasion, Grading, Adj CT
Ferrone et al	2008	USA	Retrospective	1983–2001	19	618	75	543	Age, Sex, Location, R, AJCC stage
Ueda et al	2009	Japan	Retrospective	1992–2006	19	140	20	120	Sex, Size, R, Venous invasion, PNI, Portal invasion, Vascular resection, AJCC stage
Ferrone et al	2012	USA	Retrospective	1985–2010	19	482	95	387	Sex, Location, AJCC stage, R, PNI, Grading
Lewis et al	2012	USA	Retrospective	2001–2011	20	424	100	324	Sex, ASA, Diabetes, AJCC stage, R, LVI, PNI, Vascular resection, Grading, Adj CT, Major morbidity, POPF, RBT
Robinson et al	2012	UK	Retrospective	2002–2009	19	134	25	109	AJCC stage, R, LVI, PNI, Grading
Nimura et al	2012	Japan	RCT*	2000–2003		101	11	90	Sex, Lymph-node involvement, R, LVI, Venous invasion, Vascular resection, Grading, RBT
Sinn et al	2013	Germany	RCT*	1998–2004		354	53	300	Sex, AJCC stage, R, Grading
Shin et al	2014	South Korea	Retrospective	2000–2007	21	528	82	446	Sex, Location, Size, Lymph-node involvement, R, LVI, PNI, Portal Invasion, Grading
Yamamoto et al	2015	Japan	Retrospective	2000–2011	21	96	20	76	Age, Sex, CEA, Ca19.9, AJCC Stage, R, PNI, Portal Invasion, Adj CT
Picozzi et al	2017	USA	Retrospective	2003–2010	21	176	54	122	Sex, Location, Size, Lymph-node involvement, R, Vascular resection, AJCC Stage, Grading, Neo CT
Nakano et al	2017	Japan	Retrospective	1995–2011	21	151	38	133	Age, Sex, BMI, Location, CEA, Ca 19.9, Lymph-node involvement, R, LVI, Venous Invasion, PNI, AJCC Stage, Neo CT, Adj CT, POPF
Nakagawa et al	2018	Japan	Retrospective	2006–2011	21	128	38	90	Age, Sex, BMI, ASA, Location, Ca19.9, AJCC stage, R, Vascular resection, Grading, Neo CT, Adj CT, Major morbidity, RBT
Kasahara et al	2019	Japan	Retrospective	2005–2013	19	104	21	83	Sex, Location, AJCC Stage, R, LVI, Venous Invasion, PNI, Portal invasion, Grading, Adj CT, POPF, RBT
Luu et al	2020	Germany	Retrospective	2007–2014	21	167	34	133	Age, Sex, BMI, ASA, Diabetes, Location, CEA, Ca19.9, AJCC stage, R, LVI, PNI, Portal Invasion, Grading, Neo CT, Adj CT, Major morbidity
Malleo et al	2021	Italy	Retrospective	2000–2015	19	1048	288	760	Age, Sex, ASA, Diabetes, Location, Ca19.9, AJCC stage, R, LVI, PNI, Vascular resection, Grading, Neo CT, Adj CT
Belfiori et al	2021	Italy	Retrospective	2009–2014	21	174	47	127	Sex, Lymph-node involvement, R, LVI, PNI, Vascular resection, Grading, Major morbidity

*No* number of patients included, *LTS* Long Term Survivors, *STS* Short Term Survivors, *Adj CT* adjuvant chemotherapy, *Neo CT* neoadjuvant chemotherapy, *BMI* Body Mass Index, *ASA* American Society of Anesthesiologists score, *RBT* Red Blood cell Transfusion, *LVI* Lymph-vascular invasion, *PNI* Perineural invasion, *R* Radical resection R0, *AJCC* American Joint Committee on Cancer stage, *POPF* Post-Operative Pancreatic Fistula

*Data extracted from 2 RCT evaluating respectively the role of lymphadenectomy, and the role in of adjuvant chemotherapy in long-term survival

**Table 2:** Meta-analysis of all outcomes of interest

Outcome of interest	No	Event rate (%) or mean (SD)		RR or MD (95%CI)	*p* Value	*I*^2^ (%)	Egger (*p* val)	Adjusted effect* RR or MD (95%CI)
		LTS	STS					
Diabetes	3	104/422 (24.6)	339/1217 (27.9)	0.90 (0.75; 1.10)	0.304	0.0	0.640	–
Head Location	8	526/725 (72.6)	2147/2697 (79.6)	0.94 (0.90; 0.99)	***0.010***	0.0	0.644	–
CEA	3	2,7 (0,9)	8,7 (9,8)	− 4.41(− 6.23; − 2.59)	** < *0.001***	99.1	0.443	–
Ca 19.9	5	186,6 (384,5)	302,9 (798,9)	− 358.09 (− 482.2; − 233.9)	** < *0.001***	98.1	***0.027***	− 66.42 (− 71.95; − 60.89)
Size < 30 mm	3	94/156 (60.3)	300/688 (43.6)	1.53 (1.14; 2.05)	***0.004***	62.3	0.148	–
T1-T2	11	424/746 (56.8)	1090/2652 (41.1)	1.44 (1.23; 1.68)	** < *0.001***	81.0	***0.005***	1.07 (1.03; 1.11)
T3-T4	10	323/736 (43.9)	1551/2557 (60.7)	0.85 (0.78; 0.93)	** < *0.001***	54.1	0.122	–
N0	16	466/981 (47.5)	1012/3588 (28.2)	1.82 (1.60; 2.09)	** < *0.001***	59.4	0.180	–
R0	18	864/1073 (80.5)	2838/4233 (67.0)	1.21 (1.15; 1.28)	** < *0.001***	63.5	***0.053***	1.11 (1.08; 1.13)
R +	17	209/1015 (20.6)	1351/4143 (32.6)	0.58 (0.51; 0.67)	** < *0.001***	5.1	0.090	–
PNI	12	582/832 (70.0)	2324/2993 (77.6)	0.88 (0.83; 0.92)	** < *0.001***	18.0	***0.001***	0.93 (0.90; 0.96)
LVI	9	394/708 (55.6)	1678/1971 (85.1)	0.80 (0.73; 0.89)	** < *0.001***	26.1	***0.010***	0.87 (0.83; 0.91)
Venous Invasion	4	31/90 (34.4)	259/426 (60.8)	0.60 (0.45; 0.80)	** < *0.001***	0.0	***0.017***	0.63 (0.48; 0.83)
Portal Invasion	5	31/218 (14.2)	337/1070 (31.5)	0.43 (0.19; 0.94)	0.035	75.7	***0.058***	0.85 (0.64; 1.13)
Vascular Resect	7	68/558 (12.2)	291/1633 (17.8)	0.80 (0.62; 1.02)	0.075	0.7	***0.030***	0.86 (0.68; 1.10)
AJCC St. I	9	197/676 (29.1)	377/2544 (14.8)	2.28 (1.87; 2.79)	** < *0.001***	33.2	0.324	–
AJCC St. II	8	385/666 (57.8)	1592/2449 (65.0)	0.94 (0.81; 1.08)	0.394	80.4	0.724	–
AJCC St. III	2	85/298 (28.5)	456/855 (53.3)	0.53 (0.44; 0.64)	** < *0.001***	0.0	-	–
AJCC St. IV	5	15/400 (3.8)	134/1222 (11.0)	0.57 (0.33; 0.99)	***0.046***	10.6	0.464	–
G1-2	13	635/909 (69.9)	1916/3271 (58.6)	1.21 (1.09; 1.34)	** < *0.001***	77.1	0.729	–
G3	13	214/909 (23.5)	1251/3271 (38.2)	0.61 (0.48; 0.78)	** < *0.001***	67.7	***0.012***	0.77 (0.69; 0.85)
Neoadj CT	4	103/418 (24.6)	236/1105 (21.4)	1.31 (0.84; 2.02)	0.232	81.5	0.499	–
Adjuvant CT	9	493/625 (78.9)	1414/1995 (70.9)	1.12 (0.99; 1.26)	0.066	80.8	0.945	–
Major morbidity	4	34/219 (15.5)	120/674 (17.8)	0.82 (0.58; 1.15)	0.246	0.0	***0.035***	0.82 (0.58; 1.15)
POPF	3	27/159 (17.0)	86/540 (15.9)	1.06 (0.69; 1.64)	0.785	7.1	0.857	–
RBT	4	31/170 (18.2)	199/587 (33.9)	0.56 (0.40; 0.79)	***0.001***	0.0	0.868	–

Bold, italic values indicate statistical significance

*LS* Long-term survivors, *STS* Short-term survivors, *SD* standard deviation, *MD* mean difference, *I*^2^ Higgins test, *RR* Risk Ratio, *MD* Mean Difference, *CI* Confidence Interval, Egger test for publication bias

*Adjusted effect for publication bias after Trim-and-Fill method. All RR were calculated LTS/STS, MD were calculated LTS-STS

### Clinical and laboratoristic prognostic factors

The Head Location of the tumor was significantly more frequent in the STS group (RR 0.94, 95% IC 0.90; 0.99). Regarding tumoral markers, both CEA and Ca 19.9 were significantly higher in the STS group (respectively MD − 4.41, 95% IC − 6.23; − 2.59 and MD − 358.1, 95% IC − 482.2; − 233.9). For Ca 19.9, there was some publication bias (Egger *p* = 0.027). After the Trim and Fill adjustment, the effect confirmed that higher marker levels were associated with a poorer prognosis (MD − 66.4, 95% IC − 71.9; − 60.9).

### Pathological prognostic factors

The size of tumors lower than 30 mm was significantly more frequent in the LTS group (RR 1.53, 95% IC 1.14; 2.05). Likewise, the T1-T2 stage was significantly associated with longer survival (RR 1.44, 95% IC 1.23; 1.68); on the contrary, the T3-T4 stage was associated with a poorer prognosis (RR 0.85, 95% IC 0.78; 0.93). For the T1-T2 stage, there was some publication bias (Egger *p* = 0.005), but the adjusted effect after the Trim and Fill method confirmed the prevalence in the LTS group (RR 1.07, 95% IC 1.03; 1.11). Regarding the N parameter, the absence of lymph node involvement (N0) was a prognostic factor for long survival (RR 1.82, 95% IC 1.60; 2.09). AJCC’s pathological stage has proven to be an important survival factor. Stage 1 was significantly more observed in the LTS group (RR 2.28 95% IC 1.87; 2.79), Stage III and IV were more frequent in the STS group (respectively RR 0.53, 95% IC 0.44; 0.64 and RR 0.57, 95% IC 0.33; 0.99). Stage II was not significant. In addition, a lower grading G1-2 was significantly associated with long-term survival (RR 1.21, 95% IC 1.09; 1.34), while a high-grade G3 was significantly associated with short-term survival (RR 0.61, 95% IC 0.48; 0.78). For G3, there was some publication bias (Egger *p* = 0.012), but the significance was confirmed also after Trim and Fill adjustment (RR 0.77, 95% IC 0.69; 0.85).

Finally, both Perineural Invasion and Limph-vascular invasion were associated to a short-term survival (respectively RR 0.88, 95% IC 0.83; 0.92 and RR 0.80, 95% IC 0.73; 0.89) and the data were confirmed after adjustment because of publication bias (respectively Egger *p* = 0.001 and *p* = 0.10), with RR 0.93 (95% IC 0.90; 0.96) and RR 0.87 (95% IC 0.83; 0.91) respectively.

### Surgical and therapeutic prognostic factors

Radical resection (R0) was significantly associated with long-term survival (RR 1.21, 95% IC 1.15; 1.28), and a positive resection margin (R +) was associated with short-term survival (RR 0.58, 95% IC 0.51; 0.67). For R0 resection, there was some publication bias (Egger *p* = 0.053), but the adjusted effect after the Trim and Fill method confirmed the prevalence in the LTS group (RR 1.11, 95% IC 1.08; 1.13). Venous invasion was significantly more frequent in the STS group (RR 0.60, 95% IC 0.45; 0.80), and the data were confirmed to be significant also after adjustment for publication bias (Egger 0.017, adjusted RR 0.63, 95% IC 0.48; 0.83). Both portal invasion and vascular resection were not significant after Trim-and-Fill adjustment for publication bias (respectively Egger *p* = 0.058 and *p* = 0.030), with an adjusted RR 0.85 (95%IC: 0.64; 1.13) and adjusted RR 0.86 (95% IC 0.68; 1.10), respectively. Perioperative transfusion of red blood cells, instead, was associated with short-term survival (RR 0.56, 95% IC 0.40; 0.79).

Finally, none of the following factors was proved significant in predicting survival: POPF (RR 1.06, 95% IC 0.69; 1.64), Major morbidity, defined as Clavien-Dindo ≥ 3 (RR 0.82, 95% IC 0.58; 1.15) [34], Neoadjuvant (RR 1.31, 95% IC 0.84; 2.02) and Adjuvant (RR1.12, 95% IC 0.99; 1.26) chemotherapy.

### Neoadjuvant and adjuvant chemotherapy role

We conducted an in-depth meta-regression (Table 3) to explore how neoadjuvant and adjuvant chemotherapy administration can influence long-term survival predictive factors.

**Table 3:** Meta regression analysis for Neoadjuvant and Adjuvant chemotherapy and predictive factors

Covariates	Neoadjuvant CT		Adjuvant CT	
	*ß*-coefficient (SE)	*p* Value	*ß*-coefficient (SE)	*p* Value
Year	− 0.22 (0.13)	0.089	0.02 (0.01)	0.158
East/West	0.72 (0.50)	0.148	0.18 (0.12)	0.142
MINORS	0.38 (0.24)	0.110	0.14 (0.08)	0.065
Age (MD)	− 0.02 (0.26)	0.937	0.10 (0.02)	** < *0.001***
Male sex (RR)	3.26 (1.52)	***0.032***	0.46 (0.63)	0.472
ASA I-II (RR)	− 9.94 (11.12)	0.371	− 0.10 (0.40)	0.796
BMI (MD)	0.93 (1.62)	0.564	− 0.39 (0.32)	0.218
T1-2 (RR)	0.33 (0.16)	***0.047***	0.19 (0.09)	***0.042***
LVI (RR)	− 7.49 (7.71)	0.331	− 0.25 (0.28)	0.378
PNI (RR)	− 8.56 (8.98)	0.340	0.13 (0.45)	0.775
AJCC Stage 1	0.47 (0.13)	** < *0.001***	0.07 (0.06)	0.242
AJCC Stage 2	− 0.92 (2.99)	0.758	− 0.05 (0.21)	0.822
N0 (RR)	0.55 (0.46)	0.238	− 0.01 (0.10)	0.935
R0 (RR)	1.00 (1.92)	0.601	− 0.46 (0.41)	0.271
G1-2 (RR)	0.14 (1.05)	0.897	− 0.52 (0.37)	0.161
G3 (RR)	0.42 (2.74)	0.879	0.38 (0.26)	0.146
Neo CT (RR)	–	–	0.05 (1.18)	0.760
Adj CT (RR)	0.76 (1.97)	0.700	–	–
Ca 19.9 (MD)	− 0.001 (0.001)	0.448	− 0.001 (0.001)	** < *0.001***

Bold, italic values indicate statistical significance

*MD* Mean Difference, *RR* Risk Ratio, *BMI* Body Mass Indexm, *T1-2* T parameter in TNM staging, *N0* N parameter in TNM staging, *R0* radical resection with negative margins, *G1-2* pathological grading, *Neo CT* Neoadjuvant chemotherapy, *Adj CT* Adjuvant chemotherapy

Specifically, for neoadjuvant chemotherapy, the meta-regression revealed that the association between neoadjuvant chemotherapy and long-term survival is modulated by specific clinic-pathological characteristics of patients and tumors.

In particular, a higher proportion of T1–T2 stage tumors (*β* = 0.33, *p* = 0.047) and Stage I tumors (*β* = 0.47, *p* < 0.001) in the long-term survivors (LTS) group compared to short-term survivors (STS) is associated with a more marked effect of neoadjuvant chemotherapy in determining long-term survival (Table 3, Supplementary S2 Fig. 7–8).

In addition, regarding adjuvant chemotherapy, the meta-regression revealed that its effectiveness in determining long-term survival is significantly influenced by T1-2 stage and Ca 19.9 levels (Table 3, Supplementary S2 Fig. 5–6). In particular, a higher proportion of T1–T2 stage tumors (*β* = 0.19, *p* = 0.042) and low Ca 19.9 levels (*β* = − 0.001, *p* < 0.001) in the long-term survivors (LTS) group compared to short-term survivors (STS) is associated with a more marked effect of adjuvant chemotherapy in determining long-term survival.

In other words, the benefit of neoadjuvant and adjuvant chemotherapy on long-term survival (greater than 5 years) appears to be particularly amplified in patients with early-stage tumors. This suggests that the combination of initial stage disease and neoadjuvant treatment may increase the likelihood of achieving long-term survival greater than 5 years.

## Discussion

Many prognostic indicators have been investigated in patients with LTS and PDAC in the existing literature. Nevertheless, owing to the high mortality linked with this neoplasm, long-term survivors constitute a restricted group, making it challenging for individual studies to reach statistical significance. This meta-analysis has enabled us to comprehensively examine the primary prognostic factors, having gained a substantial sample size (5412 patients, including 1097 long-term survivors).

Regarding clinical factors, our study reaffirms the association between high levels of Ca 19–9 and a poorer prognosis (*p* < 0.001) [35, 36]. This supports the concept of biological borderline resectable, introduced recently [37], and suggests a potential for stratifying patients preoperatively based on tumor biology. In contrast, the role of CEA is more contentious [35, 36]. Our meta-analysis demonstrates that elevated CEA levels significantly correlate with poor survival (*p* < 0.001).

Concerning pathological factors, for tumor size (T parameter), various size thresholds have been proposed. Garcea et al. [38] reported that a cut-off of 20 mm had the greatest impact on overall survival. In our meta-analysis, we establish that tumors smaller than 30 mm favorably impact prognosis (*p* = 0.004).

Concerning the N parameter, our study demonstrates that the absence of lymph-node involvement is associated with longer survival (*p* < 0.001). Furthermore, the ‘lymph-node ratio’ has been shown to predict survival [40, 41].

Tumor grade also plays a significant role in survival. Well-differentiated tumors are associated with longer survival [38, 39], a finding corroborated in our study (*p* < 0.001).

The absence of PNI and LVI remains a debated prognostic factors in the literature [28, 30]. However, our results demonstrate that PNI and LVI are associated with poor prognosis (*p* < 0.001).

As for therapeutic factors, our meta-analysis underscores the role of surgical effectiveness and particularly the status of surgical margins after resection (*p* < 0.001). Richter et al. [42] identified the R0 resection margin as the sole post-surgical prognostic factor. In our previous study [43], we suggested that the superior mesenteric artery margin appears most critical in defining both R status and disease-free survival among all histologically evaluated resection margins.

However, contrary to the recent literature evidence, neoadjuvant chemotherapy did not emerge as a predictive factor for LTS (*p* = 0.232). Similarly, there was no statistical difference between patients receiving adjuvant chemotherapy and those who did not (*p* = 0.066). It is essential to interpret our findings in the context of the evolving therapeutic strategies for PDAC. Our meta-analysis mainly includes studies whose data collection periods extend up to the early 2010 s (as highlighted in Table 1), preceding the widespread use of more effective chemotherapy regimens such as FOLFIRINOX and multi-agent chemotherapy in the adjuvant and neoadjuvant settings, which have been shown to improve overall survival in PDAC. Despite these general improvements in median survival, the clinicopathological factors we identified as predictors of long-term survival (e.g., grading, staging, etc.) appear to be robust. This suggests that, while current literature shows that modern treatments enhance prognosis [44–48] (although this was not reflected in our meta-analysis due to the largely pre-2010 patient cohorts), the biological mechanisms and histopathological characteristics of the disease that allow a subset of patients to become long-term survivors remain substantially unchanged.

To better investigate the impact of neoadjuvant and adjuvant chemotherapy, we performed a meta-regression (Table 3) to explore how chemotherapy administration could influence long-term survival predictive factors. Although the nature of the available data did not allow a direct comparison of long-term survival (LTS) predictive factors between patient cohorts treated upfront and those receiving neoadjuvant therapy, the meta-regression results demonstrate that the beneficial impact of neoadjuvant chemotherapy on long-term survival is particularly influenced by the presence of early-stage tumors (T1–T2 and Stage I). Similarly, the beneficial effect of adjuvant chemotherapy on long-term survival is significantly modulated by early T-stage tumors and lower Ca 19.9 levels. These findings suggest that patients with early-stage disease may derive a notably greater prognostic benefit from both neoadjuvant and adjuvant chemotherapy, which plays a crucial role in achieving long-term survival in this population. Although chemotherapy has proven efficacy even in more advanced stages, the potential to achieve long-term survival in these patients remains intrinsically limited, likely due to higher tumor burden and the biological aggressiveness of advanced disease, which continues to represent an unfavorable prognostic factor. In conclusion, while some predictive factors remain significant regardless of chemotherapy administration, others have their effects amplified by neoadjuvant or adjuvant chemotherapy. These are primarily positive prognostic factors, whose influence is enhanced by chemotherapy, suggesting that their presence in combination with treatment increases the likelihood of surviving beyond five years. Considering the limitations and based on available data—mostly from studies prior to the FOLFIRINOX or Gemcitabine-Nab-Paclitaxel era, involving few patients undergoing chemotherapy and limited chemotherapy data—it is challenging to make further definitive predictions. The key takeaway is that long-term survival is determined by certain positive prognostic factors intrinsic to the disease, such as low stage, where chemotherapy (both adjuvant and neoadjuvant) seems to play a vital supporting role to help patients achieve it. Nonetheless, patients with unfavorable prognostic characteristics could also benefit from chemotherapy, although it is less likely to result in long-term survival [44–48].

This study has some limitations. Firstly, many of the included studies had extended durations. Secondly, most of the studies in our analysis focused on patients diagnosed before 2012. Most of the data derive from patient cohorts treated before the introduction and widespread adoption of modern chemotherapy regimens (e.g., FOLFIRINOX), which have considerably improved outcomes in resectable PDAC [44–48]. Although this meta-analysis provides a robust overview of predictive factors for long-term survival based on historically available data, we were unable to perform a meaningful subgroup analysis due to the insufficient number of eligible studies with patients treated exclusively in this “new era”. Thirdly, the absence of information about the chemotherapy regimens used in most of the included studies limited our ability to analyze the effects of various treatment protocols, which can be modified to influence patient prognosis. Fourthly, the lack of genetic-molecular and laboratory data in the included studies prevented us from assessing their potential impact on long-term survival. Fifthly, another significant limitation of the present meta-analysis is the lack of detailed data on pancreatic cancer recurrence. The primary studies rarely reported information regarding the timing, anatomical location of recurrence, or specific treatments administered for recurrent disease. The availability of such data would be crucial for better understanding the impact of recurrence on long-term survival and for identifying prognostic factors at this stage of the disease, representing a key area for future research. Finally, most of the prognostic factors identified in this meta-analysis are only available after surgical resection. Therefore, while the study effectively stratifies prognostic indicators for long-term survival, it does not provide actionable preoperative tools to guide treatment strategy. This limits the immediate clinical applicability of the findings and underscores the primarily descriptive nature of the analysis.

Despite these limitations and the descriptive nature of this analysis, the large pooled sample size—uncommon in studies on long-term survivors of PDAC—has allowed us to confirm with greater certainty the prognostic value of several clinicopathological factors. This quantitative validation strengthens existing evidence and provides a more solid foundation for future research, including genetic and molecular factors [49].

## Supplementary Information

Below is the link to the electronic supplementary material.

Supplementary file1 (PDF 8290 KB)

Supplementary file2 (PDF 1022 KB)

## Data Availability

All data analyzed in this study are from previously published studies, which are cited in the reference list. No new data were collected by the authors.

## Declarations

### Registration and protocol

PROSPERO 2023 CRD42023472753.

## References

[1] Rahib L, Smith BD, Aizenberg R et al (2014) Projecting cancer incidence and deaths to 2030: the unexpected burden of thyroid, liver, and pancreas cancers in the United States. Cancer Res 74:2913–2921 24840647 10.1158/0008-5472.CAN-14-0155

[2] Mizrahi JD, Surana R, Valle JW et al (2020) Pancreatic cancer. Lancet 395(10242):2008–2020 32593337 10.1016/S0140-6736(20)30974-0

[3] Barhli A, Cros J, Bartholin L et al (2018) Prognostic stratification of resected pancreatic ductal adenocarcinoma: past, present and future. Dig Liver Dis 50(10):979–990 30205952 10.1016/j.dld.2018.08.009

[4] Neuzillet C, Tijeras-Raballand A, Bourget P et al (2015) State of the art and future directions of pancreatic ductal adenocarcinoma therapy. Pharmacol Ther 155:80–104 26299994 10.1016/j.pharmthera.2015.08.006

[5] Higgins JPTGS (2011) Cochrane handbook for systematic reviews of interventions. The Cochrane Collaboration, London

[6] Schardt C, Adams MB, Owens T et al (2007) Utilization of the PICO framework to improve searching PubMed for clinical questions. BMC Med Inform Decis Mak 7:16 17573961 10.1186/1472-6947-7-16 PMC1904193

[7] Page MJ, McKenzie JE, Bossuyt PM et al (2021) The PRISMA 2020 statement: an updated guideline for reporting systematic reviews. PLoS Med. 10.1371/journal.pmed.1003583 33780438 10.1371/journal.pmed.1003583 PMC8007028

[8] D’Ambra V, Ingaldi C, Ricci C et al (2025) Pancreatic cancer and long survivors: a survey of Italian society of oncological surgery (SICO). Update Surg 77(1):57–64 10.1007/s13304-024-02039-3 39589628

[9] Slim K, Nini E, Forestier D et al (2003) Methodological index for non-randomized studies (minors): development and validation of a new instrument. ANZ J Surg 73(9):712–716 12956787 10.1046/j.1445-2197.2003.02748.x

[10] Mantel N, Haenszel W (1959) Statistical aspects of the analysis of data from retrospective studies of disease. J Natl Cancer Inst 22:719–748 13655060

[11] Higgins JP, Thompson SG (2002) Quantifying heterogeneity in a meta-analysis. Stat Med 21:1539–1558 12111919 10.1002/sim.1186

[12] Higgins JP, Thompson SG (2004) Controlling the risk from spurious findings from meta-regression. Stat Med 23:1663–1682 15160401 10.1002/sim.1752

[13] Egger M, Davey Smith G, Schneider M (1997) Bias in meta-analysis detected by a simple, graphical test. BMJ 315:629–634 9310563 10.1136/bmj.315.7109.629 PMC2127453

[14] Duval S, Tweedie R (2000) Trim and fill: a simple funnel-plot-based method of testing and adjusting for publication bias in meta-analysis. Biometrics 56(2):455–463 10877304 10.1111/j.0006-341x.2000.00455.x

[15] Mosca F, Giulianotti PC, Balestracci T et al (1997) Long-term survival in pancreatic cancer: pylorus-preserving versus Whipple pancreatoduodenectomy. Surgery 122(3):553–566 9308613 10.1016/s0039-6060(97)90128-8

[16] Ahmad NA, Lewis JD, Ginsberg GG et al (2001) Long term survival after pancreatic resection for pancreatic adenocarcinoma. Am J Gastroenterol 96(9):2609–2615 11569683 10.1111/j.1572-0241.2001.04123.x

[17] Schnelldorfer T, Ware AL, Sarr MG et al (2008) Long-term survival after pancreatoduodenectomy for pancreatic adenocarcinoma: is cure possible? Ann Surg 247(3):456–462 18376190 10.1097/SLA.0b013e3181613142

[18] Ferrone CR, Brennan MF, Gonen M et al (2008) Pancreatic adenocarcinoma: the actual 5-year survivors. J Gastrointest Surg 12(4):701–706 18027062 10.1007/s11605-007-0384-8

[19] Ueda M, Endo I, Nakashima M et al (2009) Prognostic factors after resection of pancreatic cancer. World J Surg 33(1):104–110 19011933 10.1007/s00268-008-9807-2

[20] Ferrone CR, Pieretti-Vanmarcke R, Bloom JP et al (2012) Pancreatic ductal adenocarcinoma: long-term survival does not equal cure. Surgery 152(3):S43–S49 22763261 10.1016/j.surg.2012.05.020 PMC3806092

[21] Lewis R, Drebin JA, Callery MP et al (2013) A contemporary analysis of survival for resected pancreatic ductal adenocarcinoma. HPB 15(1):49–60 23216779 10.1111/j.1477-2574.2012.00571.x PMC3533712

[22] Robinson SM, Rahman A, Haugk B et al (2012) Metastatic lymph node ratio as an important prognostic factor in pancreatic ductal adenocarcinoma. Eur J Surg Oncol 38(4):333–339 22317758 10.1016/j.ejso.2011.12.020

[23] Nimura Y, Nagino M, Takao S et al (2012) Standard versus extended lymphadenectomy in radical pancreatoduodenectomy for ductal adenocarcinoma of the head of the pancreas: long-term results of a Japanese multicenter randomized controlled trial. J Hepatobiliary Pancreat Sci 19(3):230–241 22038501 10.1007/s00534-011-0466-6

[24] Sinn M, Striefler JK, Sinn BV et al (2013) Does long-term survival in patients with pancreatic cancer really exist? Results from the CONKO-001 study. J Surg Oncol 108(6):398–402 24038103 10.1002/jso.23409

[25] Shin SH, Kim SC, Hong SM et al (2014) Can statistically determind prognostic factors predict the long-term survival of patients with pancreatic ductal adenocarcinoma following surgical resection?: Clinicopathological analysis of 82 long-term survivors. Pancreas 43(4):571–577 24681875 10.1097/MPA.0000000000000063

[26] Yamamoto T, Yagi S, Kinoshita H et al (2015) Long-term survival after resection of pancreatic cancer: a single-center retrospective analysis. World J Gastroenterol 21(1):262–268 25574100 10.3748/wjg.v21.i1.262 PMC4284344

[27] Picozzi VJ, Oh SY, Edwards A et al (2017) Five-year actual overall survival in resected pancreatic cancer: a contemporary single-institution experience from a multidisciplinary perspective. Ann Surg Oncol 24(6):1722–1730 28054192 10.1245/s10434-016-5716-z

[28] Nakano Y, Kitago M, Shinoda M et al (2017) Clinical predictive factors of long-term survival after curative resection of pancreatic cancer: a retrospective study. Cancer Med 6(10):2278–2286 28925039 10.1002/cam4.1178 PMC5633589

[29] Nakagawa K, Akahori T, Nishiwada S et al (2018) Prognostic factors for actual long-term survival in the era of multidisciplinary treatment for pancreatic ductal adenocarcinoma. Langenbecks Arch Surg 403(6):693–700 30218193 10.1007/s00423-018-1709-7

[30] Kasahara N, Noda H, Kakizawa N et al (2019) A lack of postoperative complications after pancreatectomy contributes to the long-term survival of patients with pancreatic cancer. Pancreatology 19(5):686–694 31253497 10.1016/j.pan.2019.06.012

[31] Luu AM, Braumann C, Belyaev O et al (2021) Long-term survival after pancreaticoduodenectomy in patients with ductal adenocarcinoma of the pancreatic head. Hepatobiliary Pancreat Dis Int 20(3):271–278 33349608 10.1016/j.hbpd.2020.12.006

[32] Malleo G, Maggino L, Lionetto G et al (2023) A dynamic analysis of empirical survival outcomes after pancreatectomy for pancreatic ductal adenocarcinoma. Surgery 173(4):1030–1038 36585320 10.1016/j.surg.2022.11.015

[33] Belfiori G, Crippa S, Francesca A et al (2021) Long-term survivors after upfront resection for pancreatic ductal adenocarcinoma: an actual 5-year analysis of disease-specific and post-recurrence survival. Ann Surg Oncol 28(13):8249–8260 34258720 10.1245/s10434-021-10401-7

[34] Dindo D, Demartines N, Clavien P (2004) Classification of surgical complications. Ann Surg 240(2):205–213 15273542 10.1097/01.sla.0000133083.54934.ae PMC1360123

[35] Hata T, Chiba K, Mizuma M et al (2022) Levels of tumor markers CEA/CA 19–9 in serum and peritoneal lavage predict postoperative recurrence in patients with pancreatic cancer. Ann Gastroenterol Surg 6(6):862–872 36338582 10.1002/ags3.12597 PMC9628216

[36] van Manen L, Groen JV, Putter H et al (2020) Stage-specific value of carbohydrate antigen 19–9 and carcinoembryonic antigen serum levels on survival and recurrence in pancreatic cancer: a single center study and meta-analysis. Cancers (Basel). 12(10):2970 33066393 10.3390/cancers12102970 PMC7602123

[37] Kato Y, Yamada S, Tashiro M et al (2019) Biological and conditional factors should be included when defining criteria for resectability for patients with pancreatic cancer. HPB (Oxford). 10.1016/j.hpb.2019.01.012 30773450 10.1016/j.hpb.2019.01.012

[38] Garcea G, Dennison AR, Pattenden CJ et al (2008) Survival following curative resection for pancreatic ductal adenocarcinoma. A systematic review of the literature. JOP 9(2):99–132 18326920

[39] Ingaldi C, D’Ambra V, Ricci C et al (2024) Clinicopathological predictive factors in long-term survivors who underwent surgery for pancreatic ductal adenocarcinoma: a single-center propensity score matched analysis. World J Surg 48(12):3001–3013 39542862 10.1002/wjs.12397 PMC11619735

[40] Luu AM, Braumann C, Belayaev O et al (2021) Long-term survival after pancreaticoduodenectomy in patients with ductal adenocarcinoma of the pancreatic head. Hepatobiliary Pancreat Dis Int 20:271–278 33349608 10.1016/j.hbpd.2020.12.006

[41] Zheng ZJ, Wang MJ, Tan CL et al (2020) Prognostic impact of lymph node status in patients after total pancreatectomy for pancreatic ductal adenocarcinoma. Medicine (Baltimore). 10.1097/md.0000000000019327 32080152 10.1097/MD.0000000000019327 PMC7034702

[42] Richter A, Niedergethmann M, Sturm JW et al (2003) Long-term results of partial pancreaticoduodenectomy for ductal adenocarcinoma of the pancreatic head: 25-year experience. World J Surg 27(3):324–329 12607060 10.1007/s00268-002-6659-z

[43] Casadei R, Ricci C, Taffurelli G et al (2018) Multicolour versus monocolour inking specimens after pancreaticoduodenectomy for periampullary cancer: a single centre prospective randomised clinical trial. Int J Surg 51:63–70 29367035 10.1016/j.ijsu.2018.01.021

[44] Schorn S, Demir IE, Reyes CM et al (2017) The impact of neoadjuvant therapy on the histopathological features of pancreatic ductal adenocarcinoma - a systematic review and meta-analysis. Cancer Treat Rev 55:96–106 28342938 10.1016/j.ctrv.2017.03.003

[45] Ghaneh P, Palmer D, Cicconi S et al (2023) Immediate surgery compared with short-course neoadjuvant gemcitabine plus capecitabine, FOLFIRINOX, or chemoradiotherapy in patients with borderline resectable pancreatic cancer (ESPAC5): a four-arm, multicentre, randomised, phase 2 trial. Lancet Gastroenterol Hepatol 8(2):157–168 36521500 10.1016/S2468-1253(22)00348-X

[46] Tempero MA, Malafa MP, Al-Hawary M et al (2021) Pancreatic adenocarcinoma, NCCN clinical practice guidelines in oncology. J Natl Compr Canc Netw 19(4):439–457 33845462 10.6004/jnccn.2021.0017

[47] Canadian Cancer Trials Group and the Unicancer-GI–PRODIGE Group, Conroy T, Hammel P, Hebbar M et al (2018) FOLFIRINOX or gemcitabine as adjuvant therapy for pancreatic cancer. N Engl J Med 379(25):2395–2406 30575490 10.1056/NEJMoa1809775

[48] Von Hoff DD, Ervin T, Arena FP et al (2013) Increased survival in pancreatic cancer with nab-paclitaxel plus gemcitabine. N Engl J Med 369(18):1691–1703 24131140 10.1056/NEJMoa1304369 PMC4631139

[49] Barhli A, Cros J, Bartholin L et al (2018) Prognostic stratification of resected pancreatic ductal adenocarcinoma: past, present, and future. Dig Liver Dis 50(10):979–990 30205952 10.1016/j.dld.2018.08.009

