# Supplementary material for: Predictive factors for long-term survival in pancreatic ductal adenocarcinoma that underwent surgery: a systematic review and meta-analysis of literature
Source: Updates Surg. 2025 Sep 1;78(2):509–18. doi: 10.1007/s13304-025-02382-z (PMC13212744; doi:10.1007/s13304-025-02382-z)
Supplement: Supplementary file 1 — Supplementary file1 (PDF 8290 KB) [file 13304_2025_2382_MOESM1_ESM.pdf]

Baseline Characteristics

Overall Effect favours Long-Term-Survivors if RR > 1 or MD > 0.

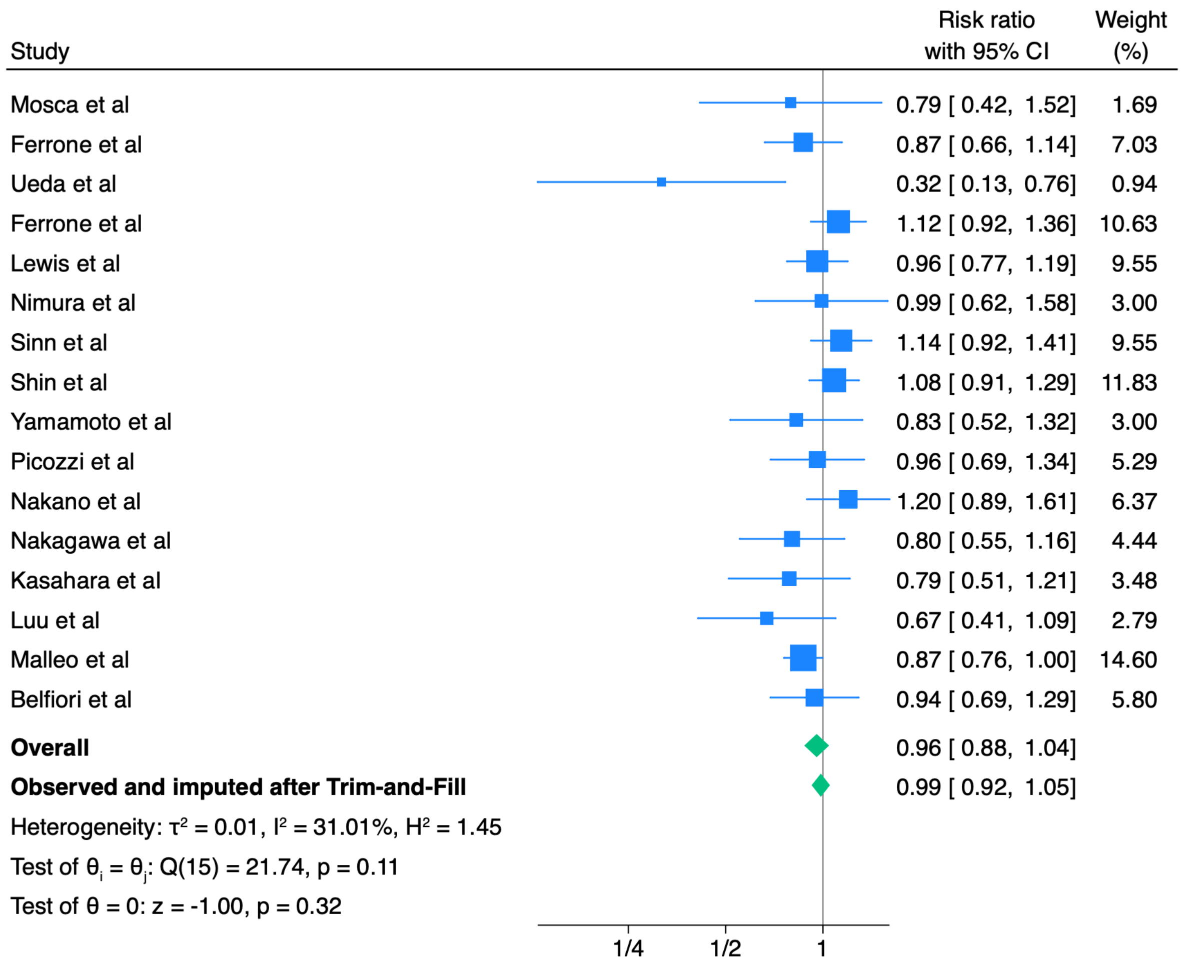

Figure 1. Male sex, forest plot.

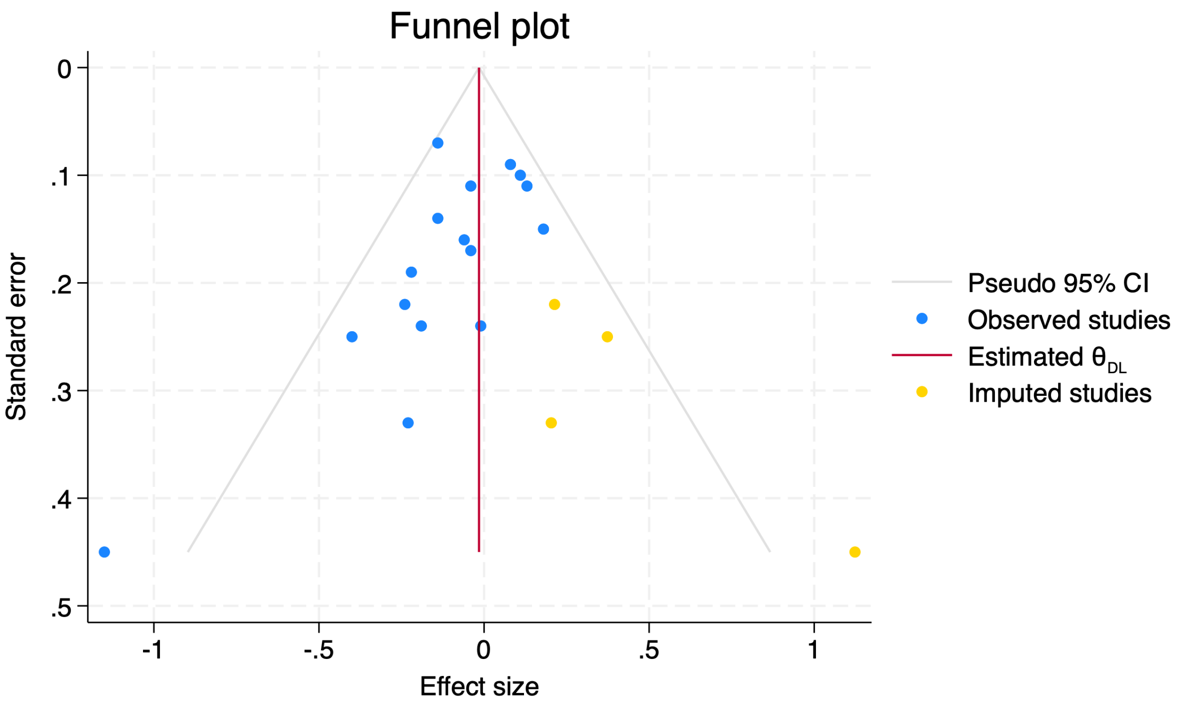

Figure 2. Male sex, funnel plot. Yellow dots represent impoted studies using Trim-and-Fill method, because the presence of publication bias.

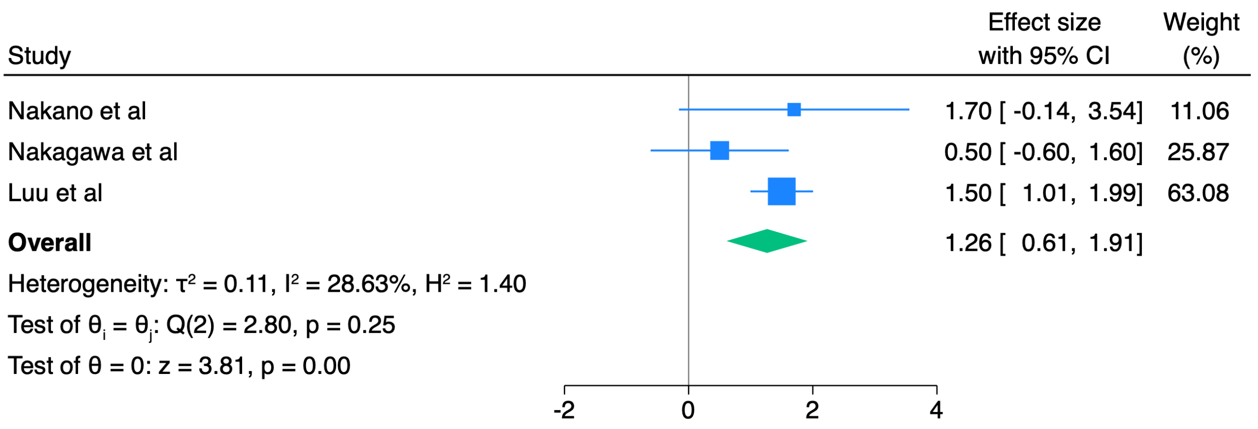

Figure 3. BMI, forest plot.

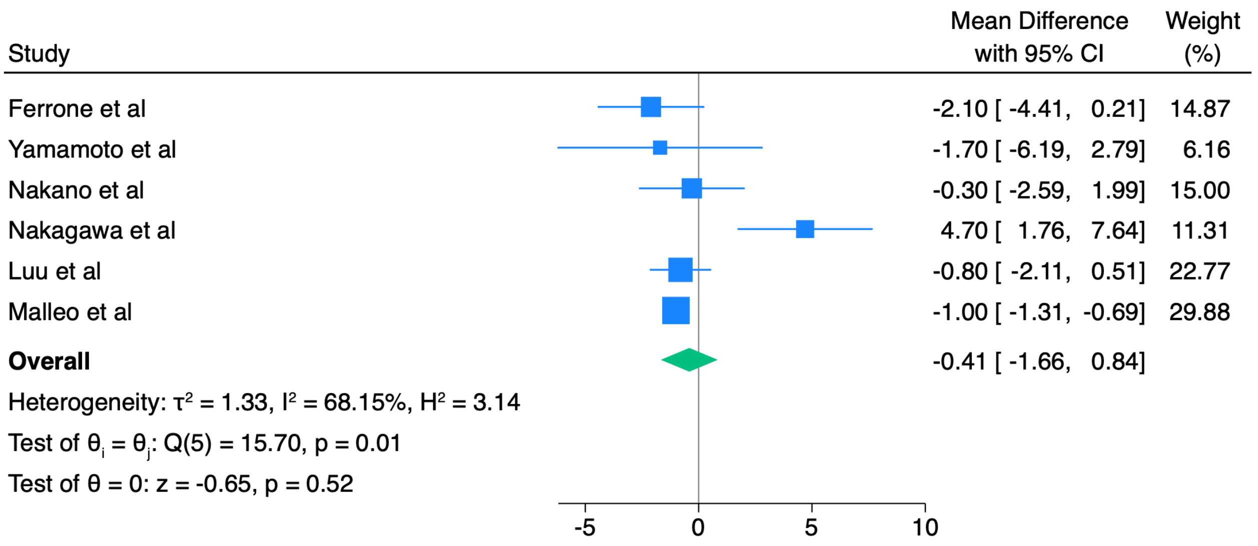

Figure 4. Age, forest plot.

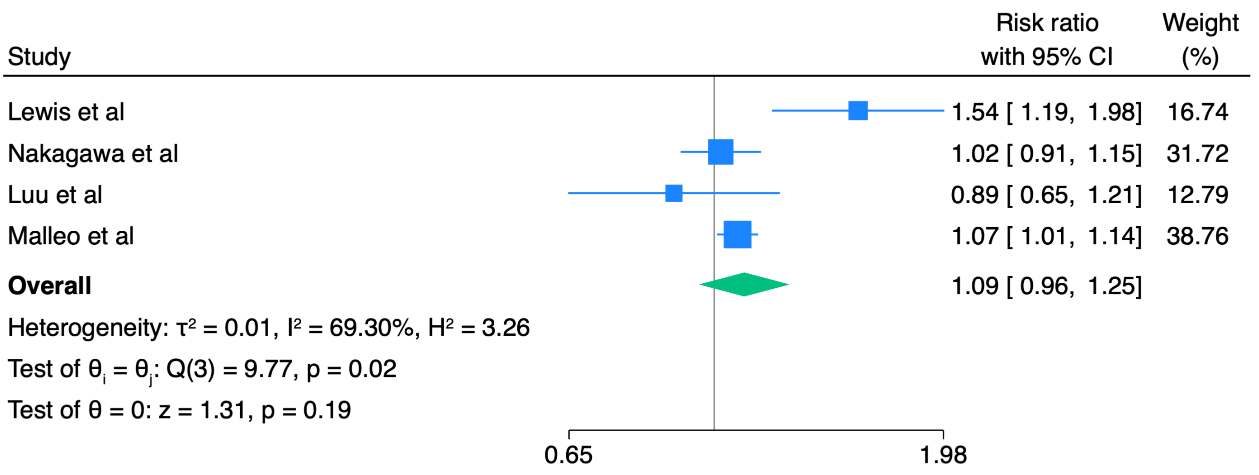

Figure 5. ASA I-II, forest plot.

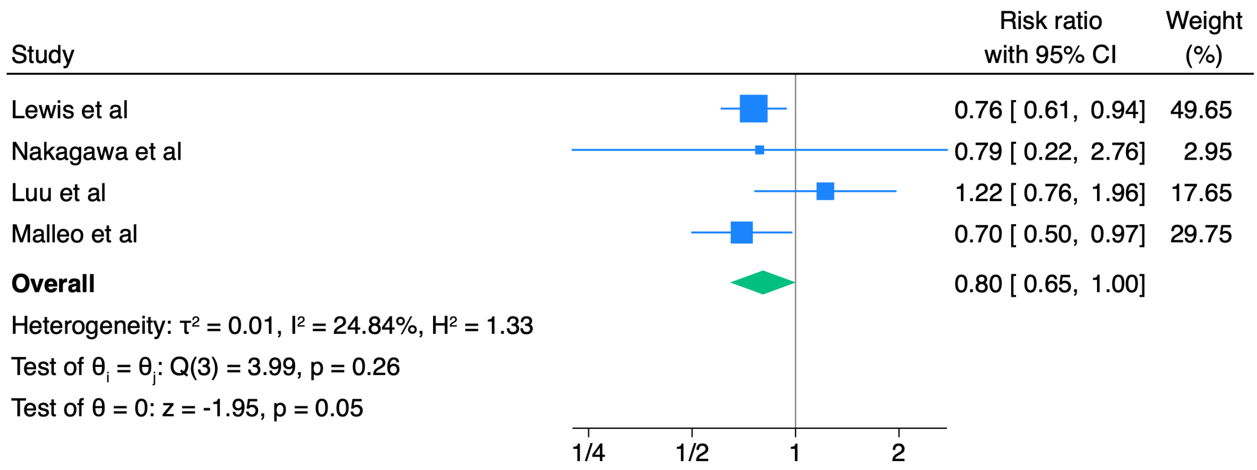

Figure 6. ASA III-IV, forest plot.

Pubblication Bias and funnel plots.

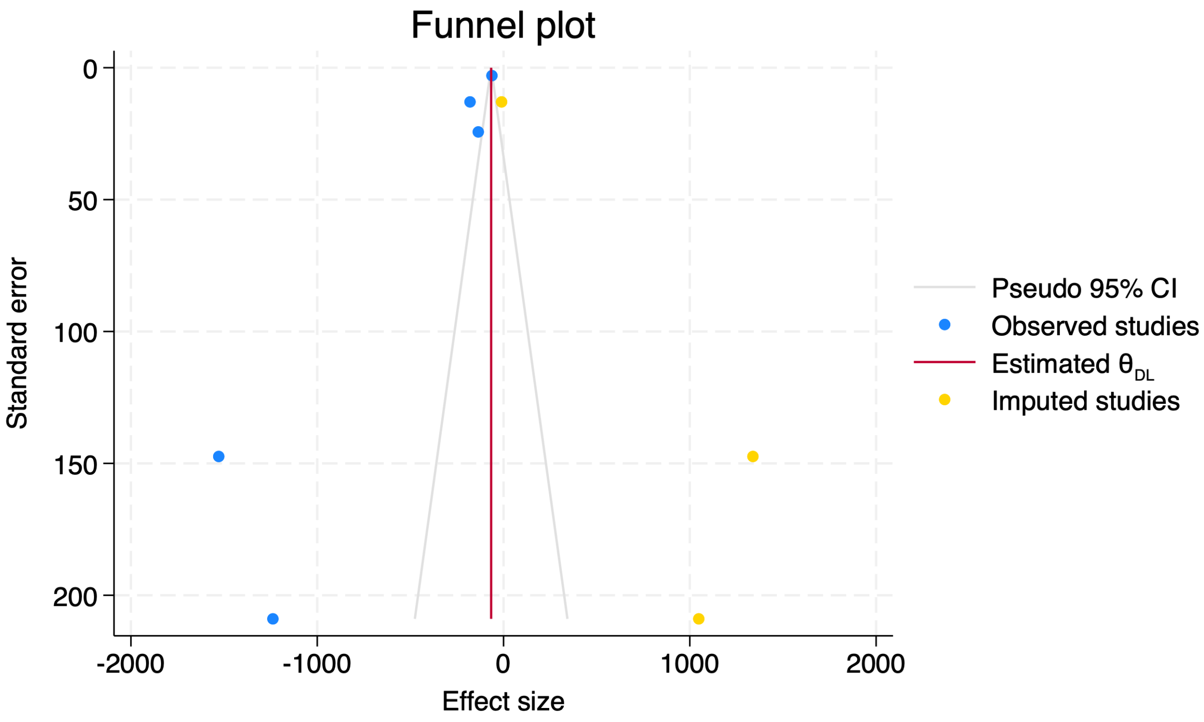

Figure 7. Ca 19.9, funnel plot. Yellow dots represent impoted studies using Trim-and-Fill method, because the presence of publication bias.

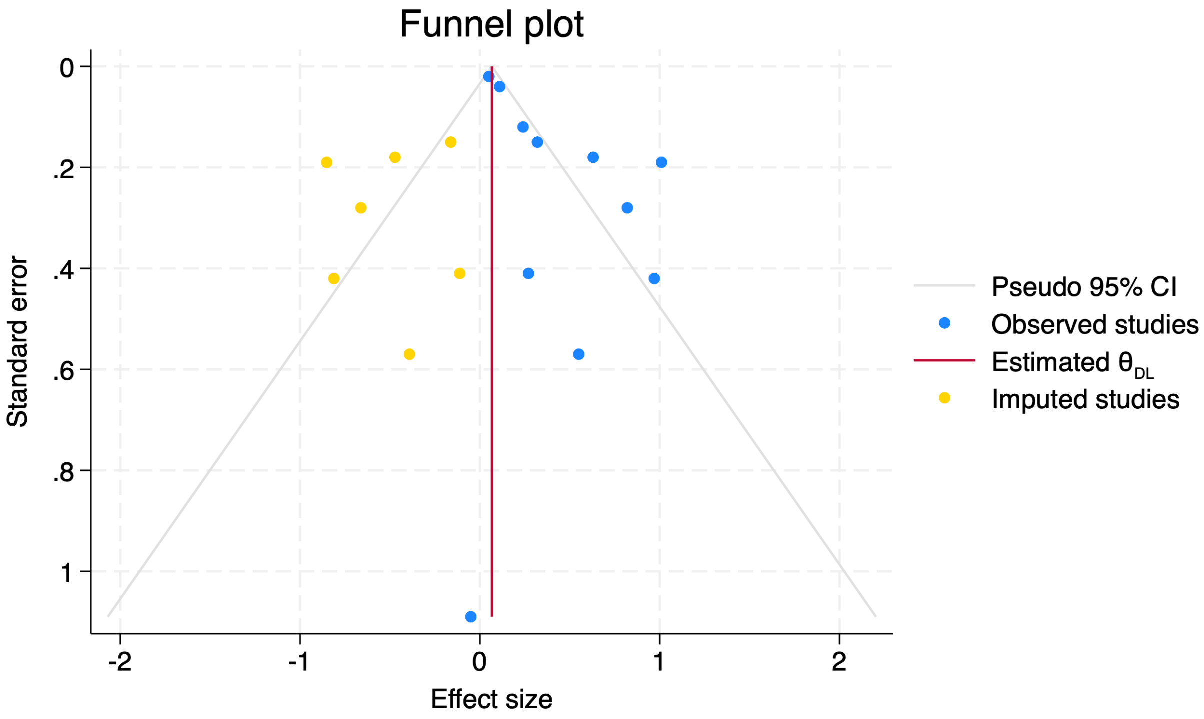

Figure 8. T1-2 stage, funnel plot. Yellow dots represent impoted studies using Trim-and-Fill method, because the presence of publication bias.

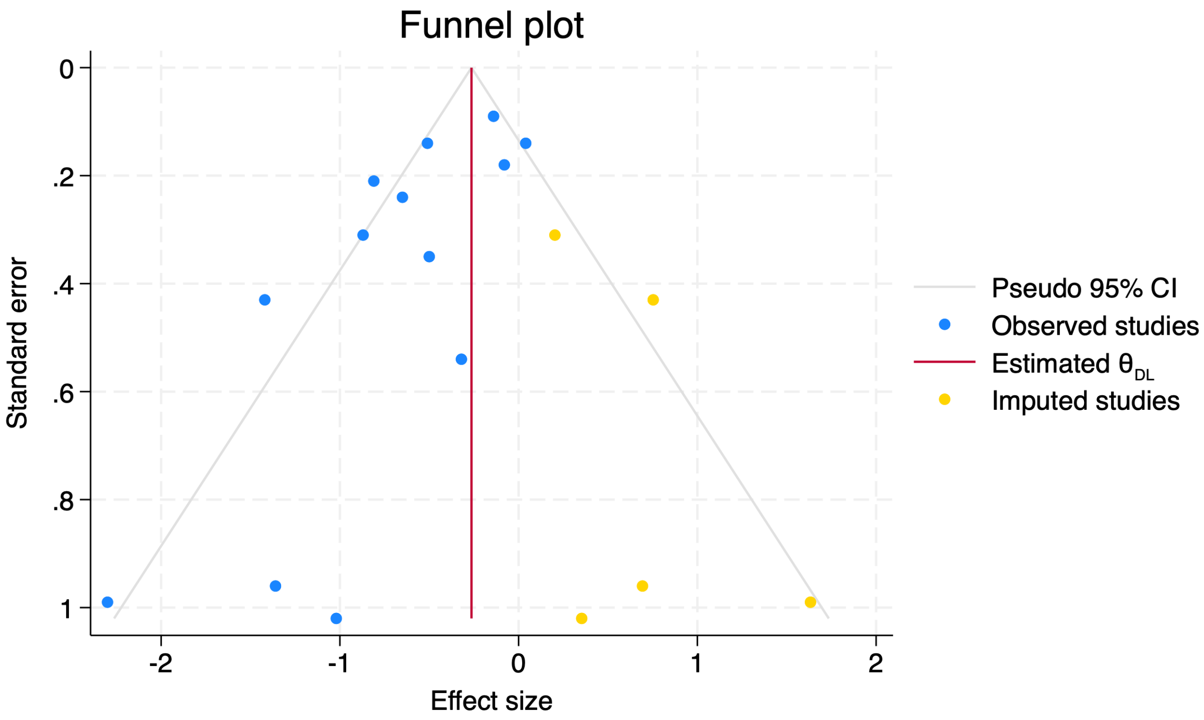

Figure 9. G3 grading, funnel plot. Yellow dots represent impoted studies using Trim-and-Fill method, because the presence of publication bias.

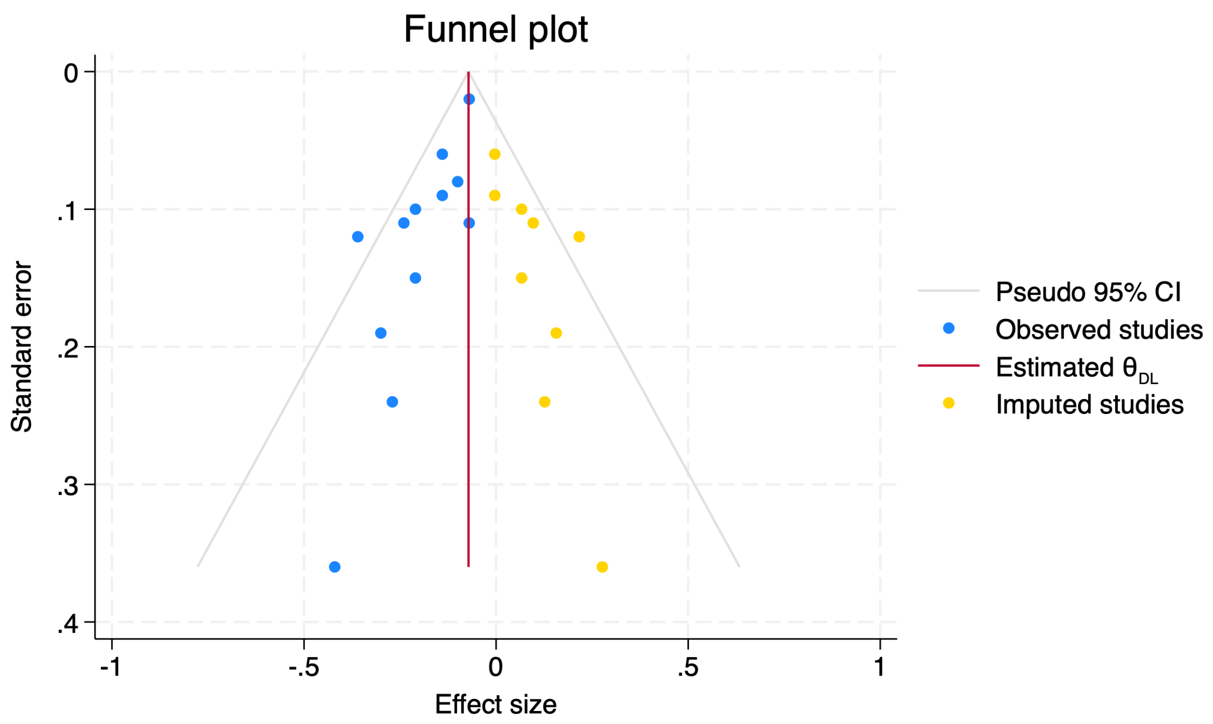

Figure 10. Perineural Invasion (PNI), funnel plot. Yellow dots represent impoted studies using Trim-and-Fill method, because the presence of publication bias.

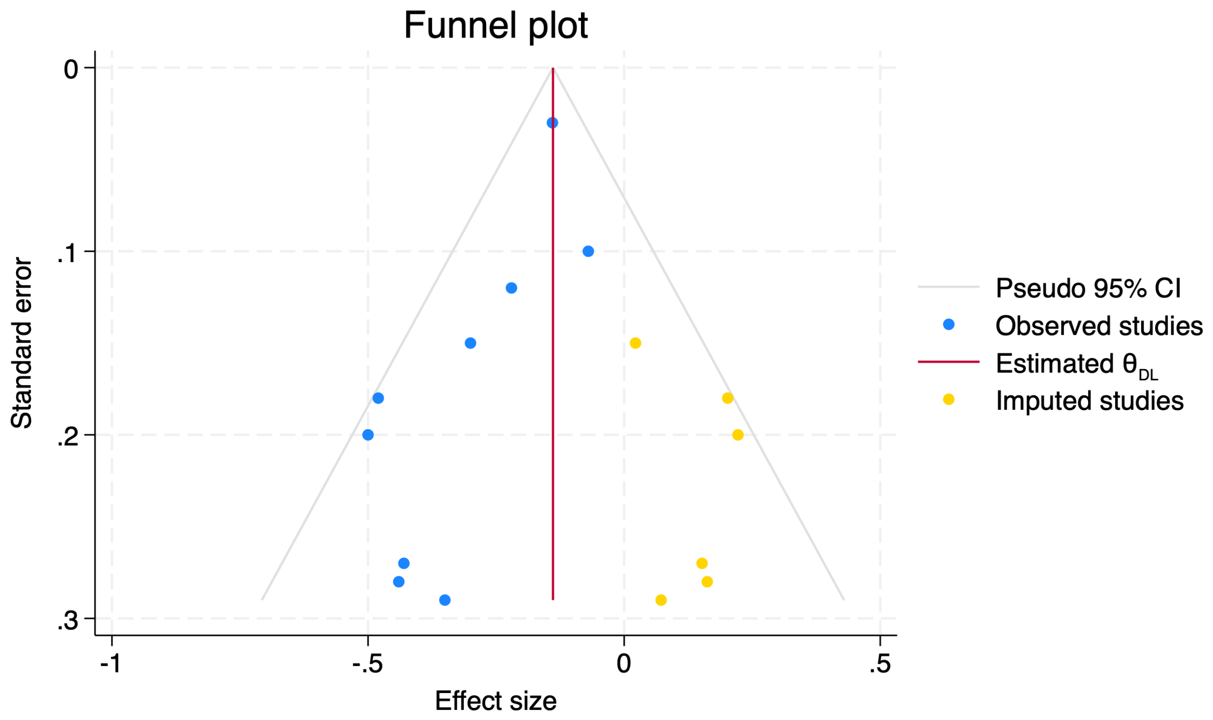

Figure 11. Limph-vascular Invasion (LVI), funnel plot. Yellow dots represent impoted studies using Trim-and-Fill method, because the presence of publication bias.

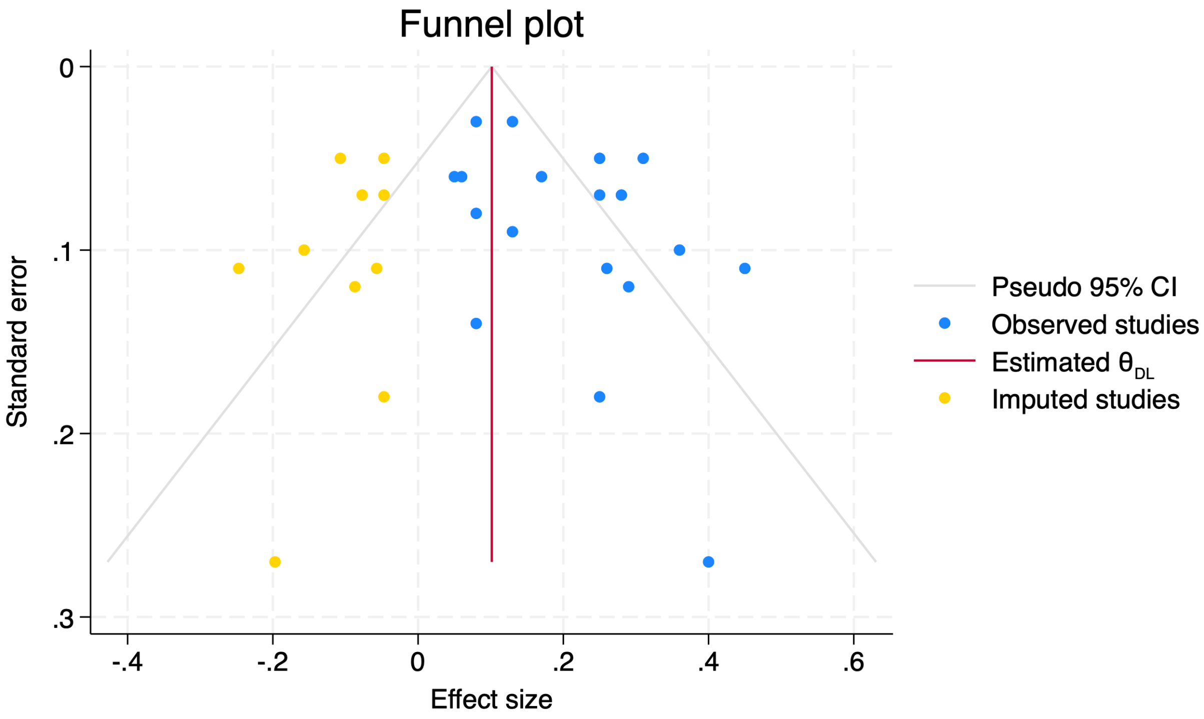

Figure 12. R0 resection, funnel plot. Yellow dots represent impoted studies using Trim-and-Fill method, because the presence of publication bias.

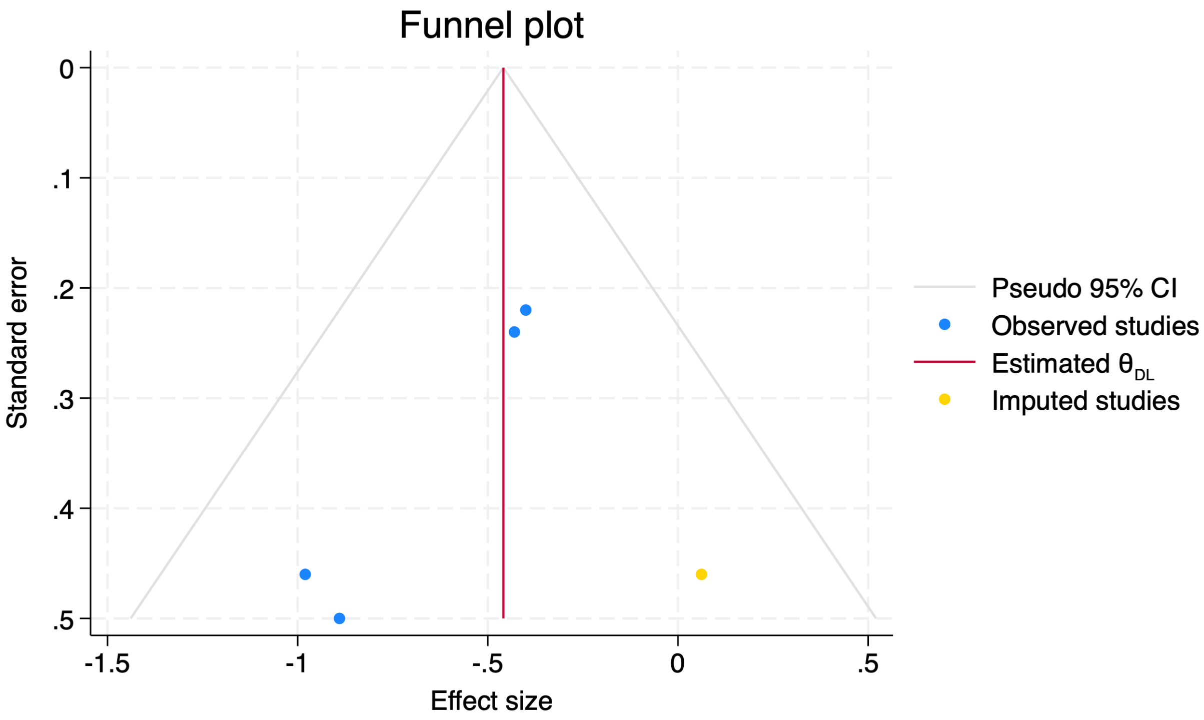

Figure 13. Venous invasion, funnel plot. Yellow dots represent impoted studies using Trim-and-Fill method, because the presence of publication bias.

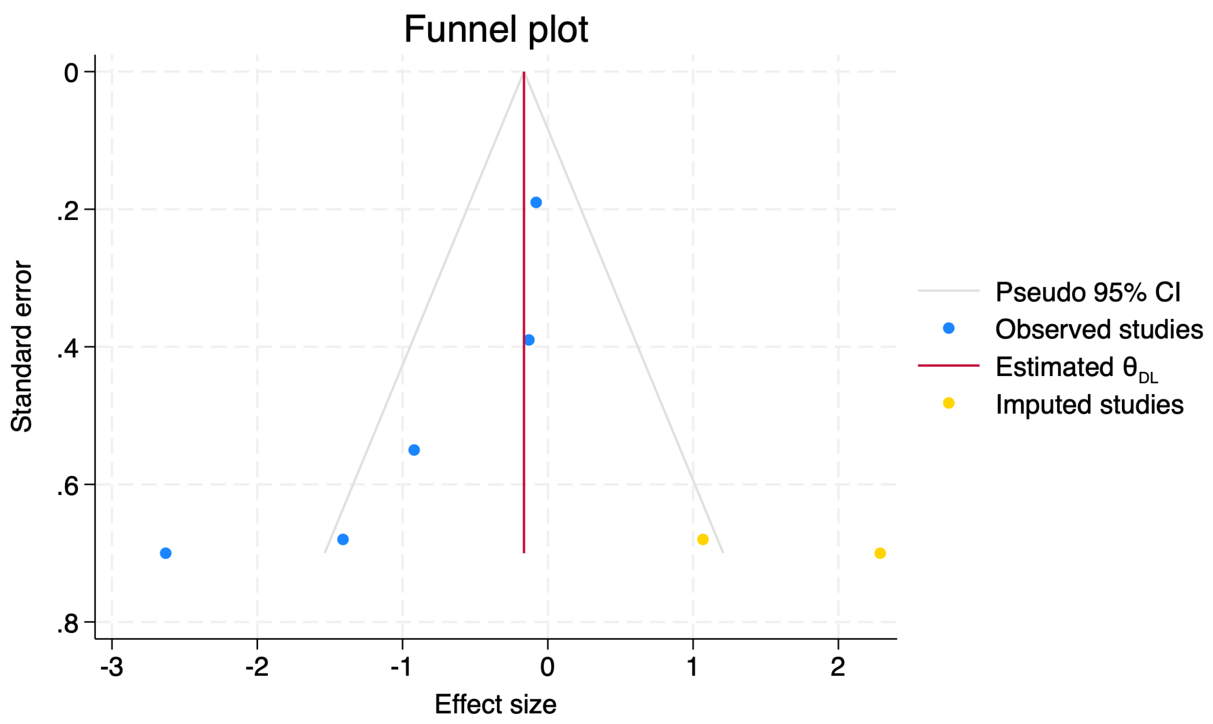

Figure 14. Portal invasion, funnel plot. Yellow dots represent impoted studies using Trim-and-Fill method, because the presence of publication bias.

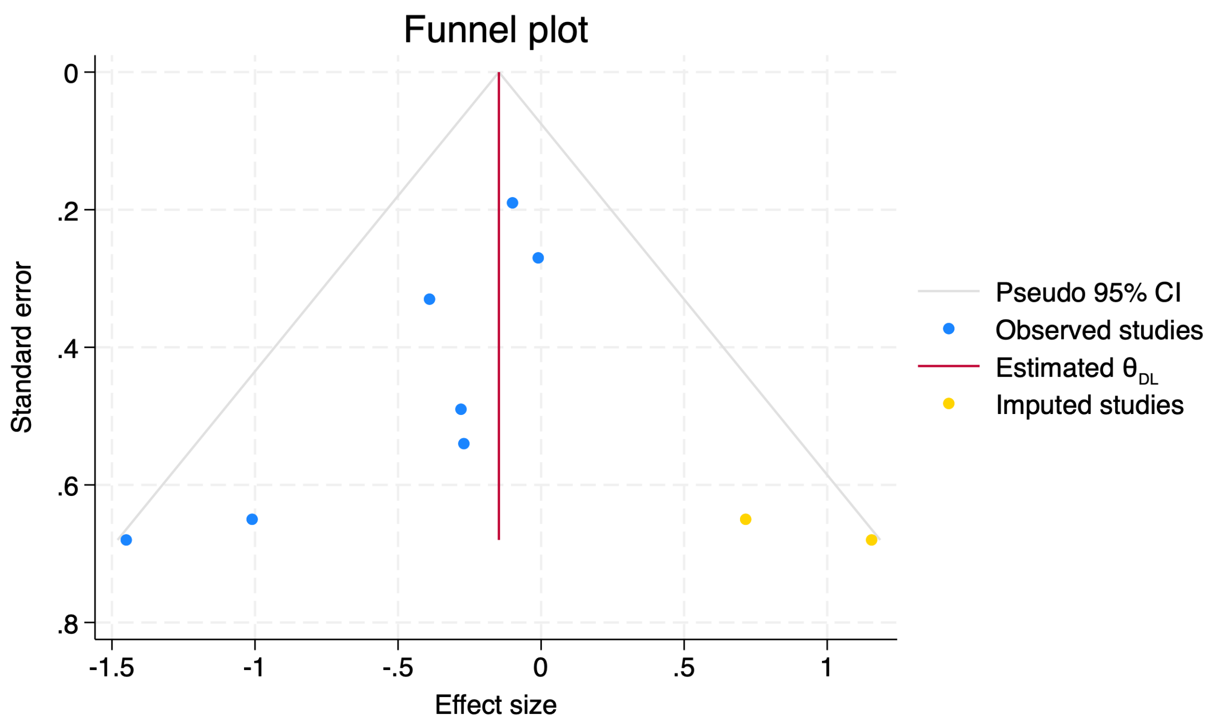

Figure 15. Vascular resection, funnel plot. Yellow dots represent impoted studies using Trim-and-Fill method, because the presence of publication bias.

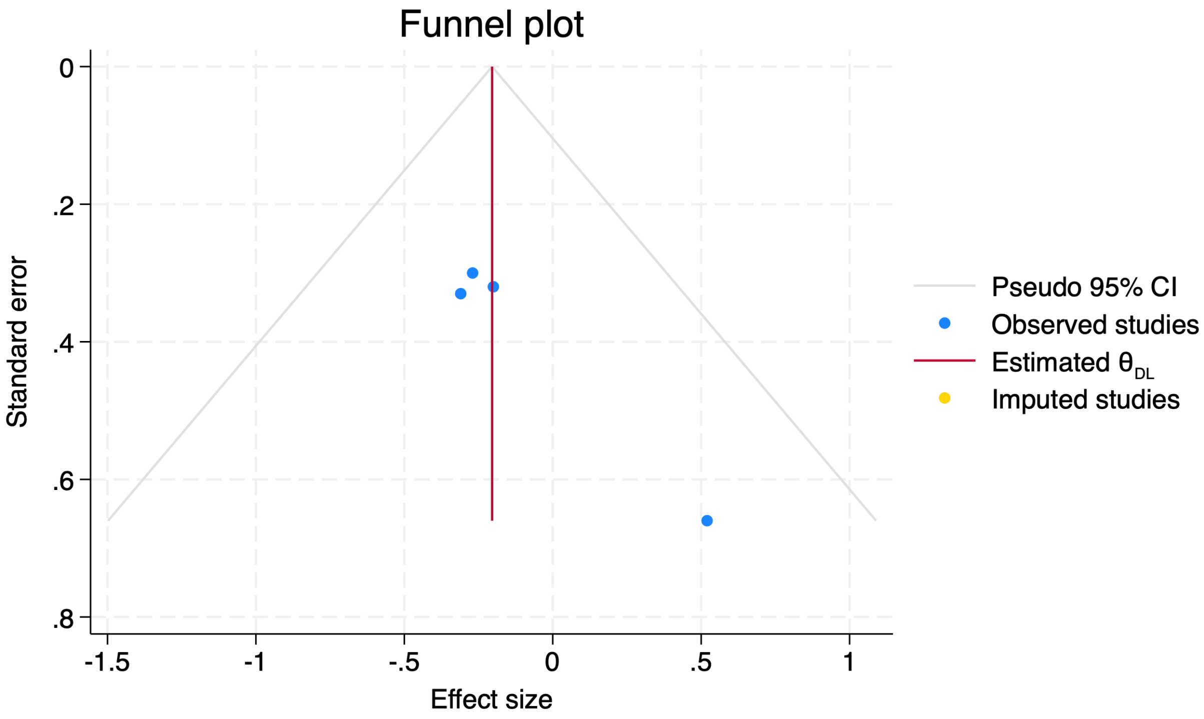

Figure 16. Major morbidity, funnel plot. Yellow dots represent impoted studies using Trim-and-Fill method, because the presence of publication bias.

Forest plot of main outcomes, clinical and laboratoristic.
Overall Effect favours Long-Term-Survivors if RR > 1 or MD > 0.

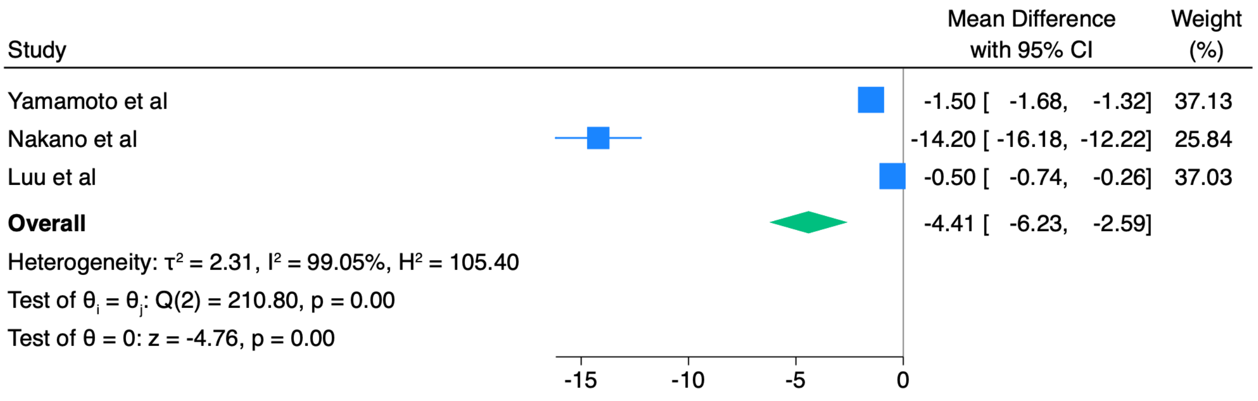

Figure 17. CEA levels, forest plot.

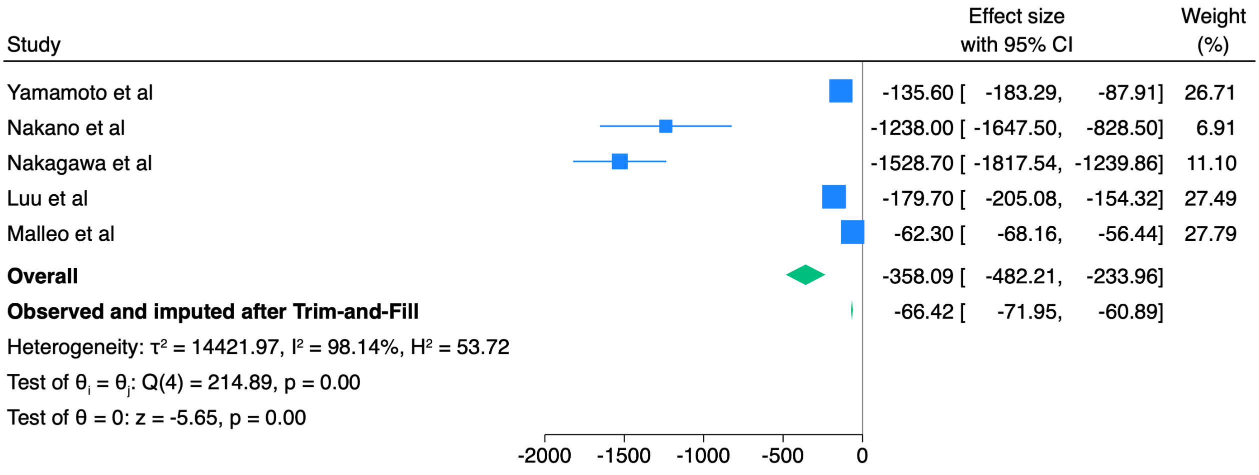

Figure 18. Ca 19.9 levels, forest plot.

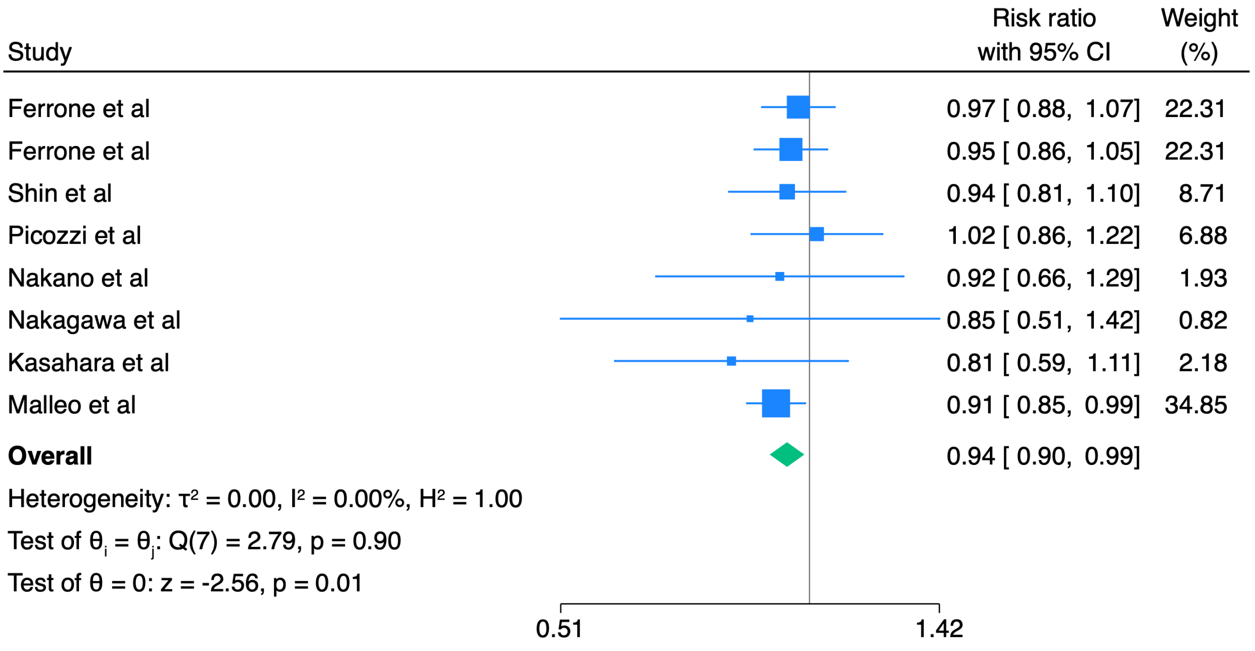

Figure 19. Head location, forest plot.

Forest plot of main outcomes, pathological.

Overall Effect favours Long-Term-Survivors if RR > 1 or MD > 0.

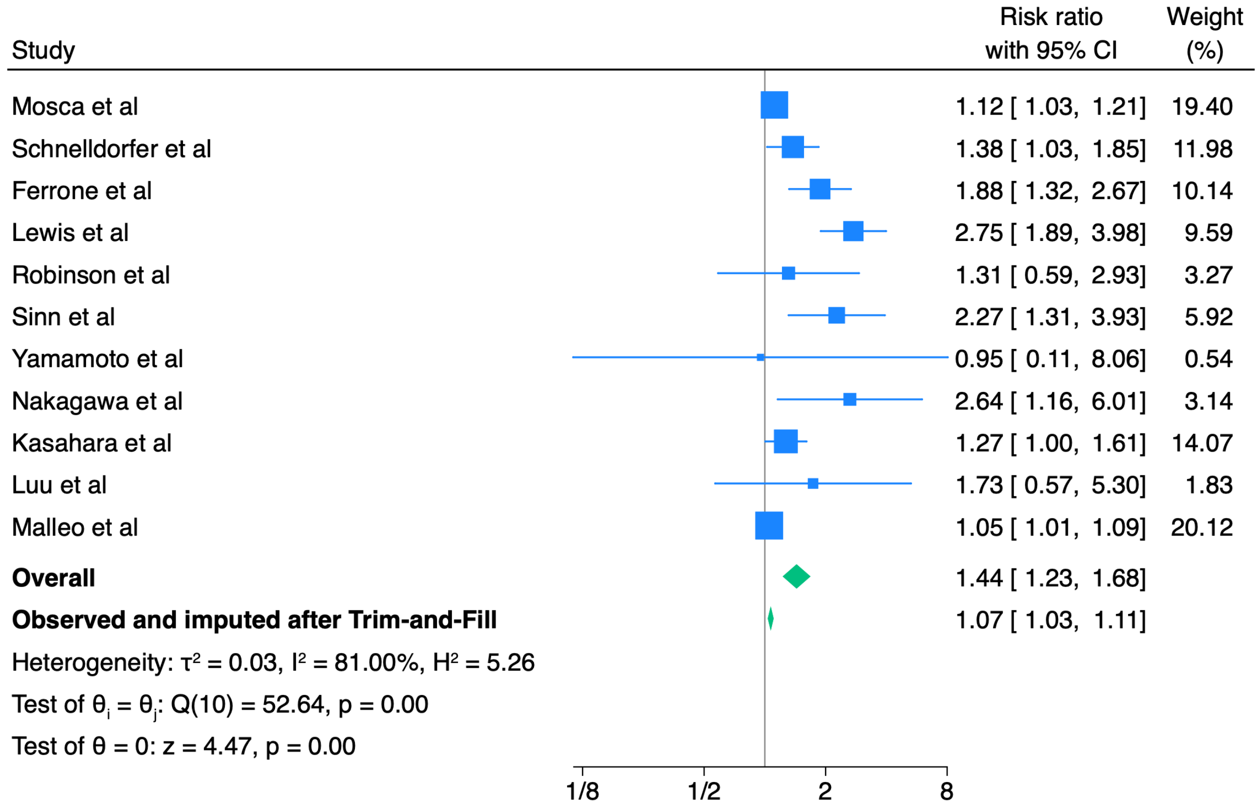

Figure 20. T1-T2 Stage, forest plot.

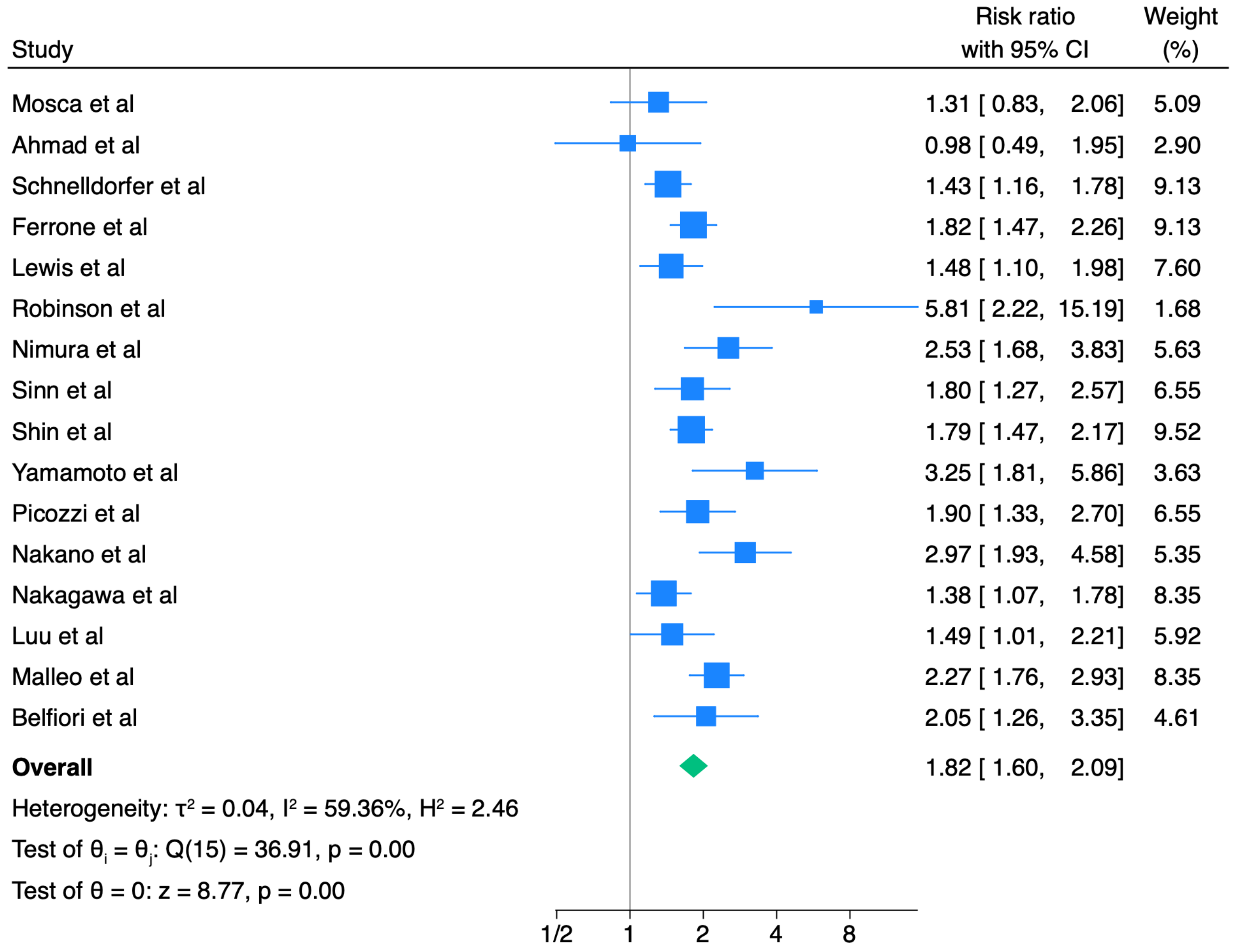

Figure 21. N0 stage, forest plot.

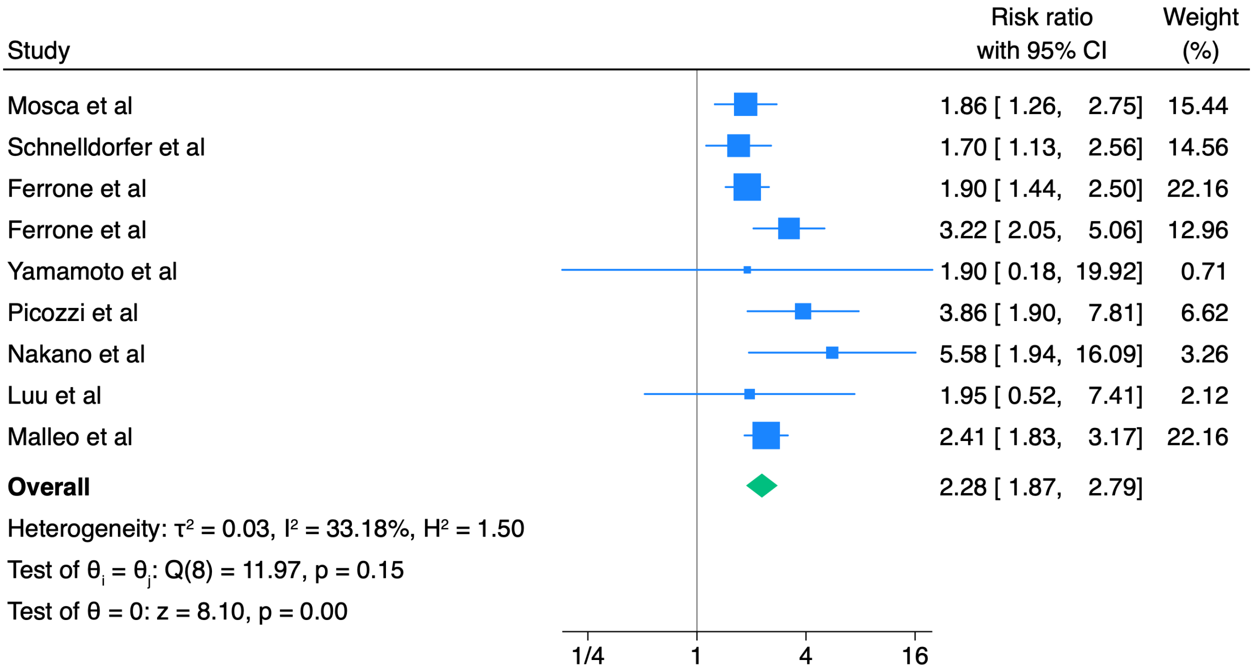

Figure 22. Stage I, forest plot.

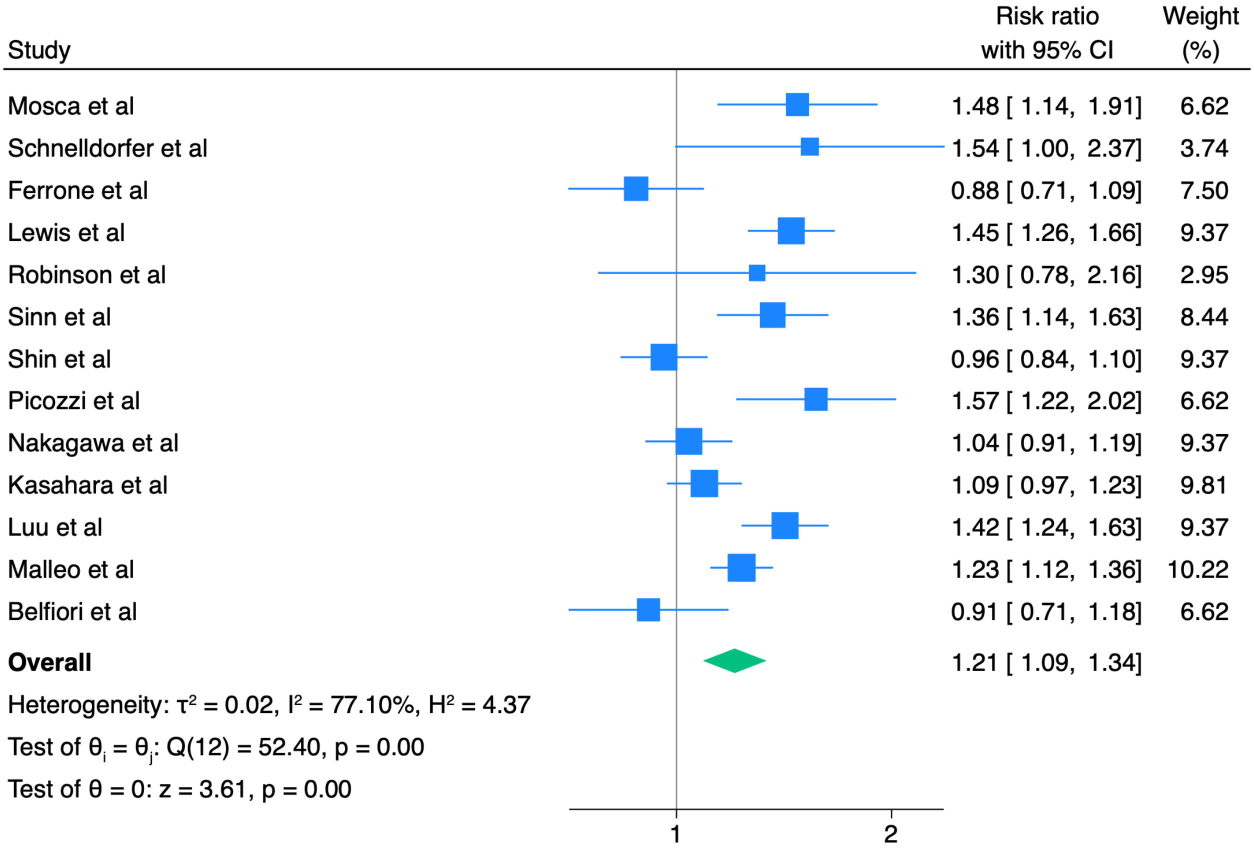

Figure 23. G1-G2 grading, forest plot.

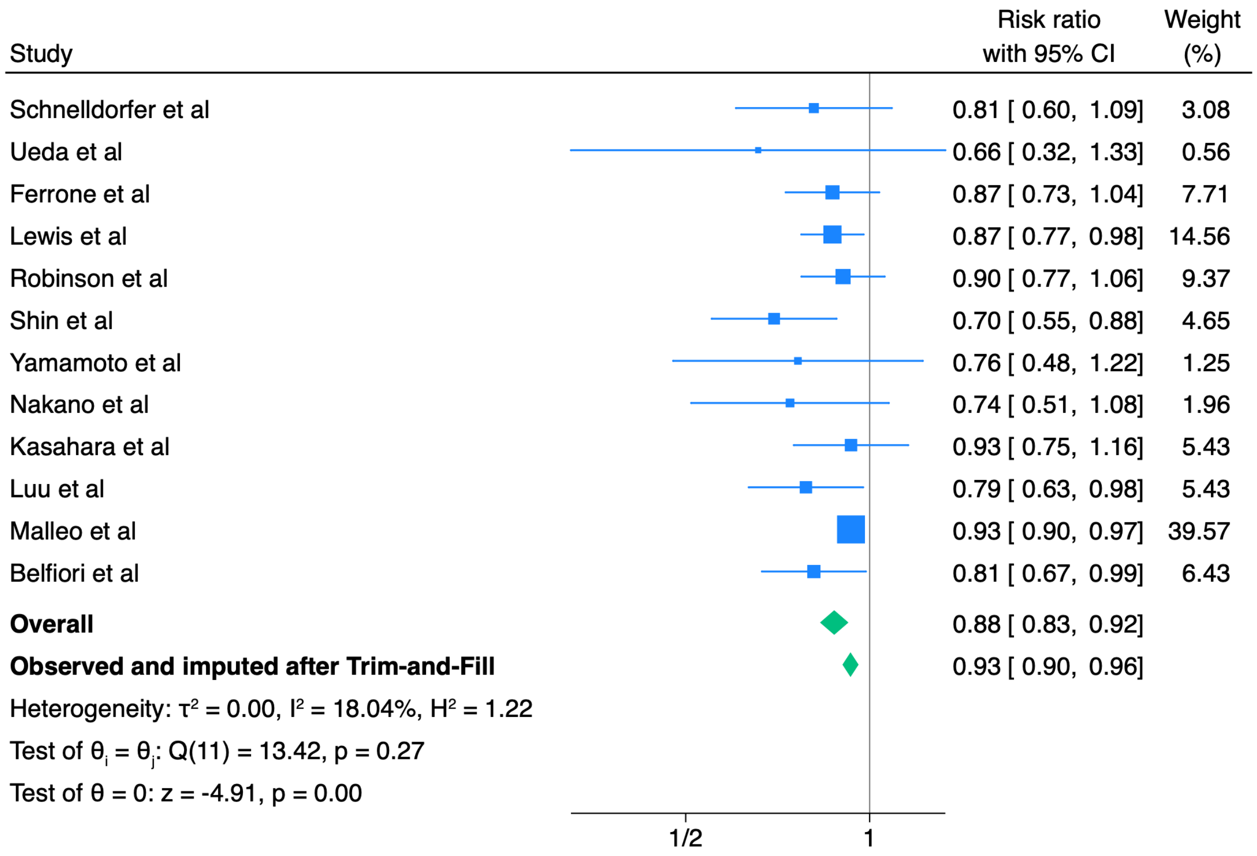

Figure 24. Peri-Neural Invasion (PNI), forest plot.

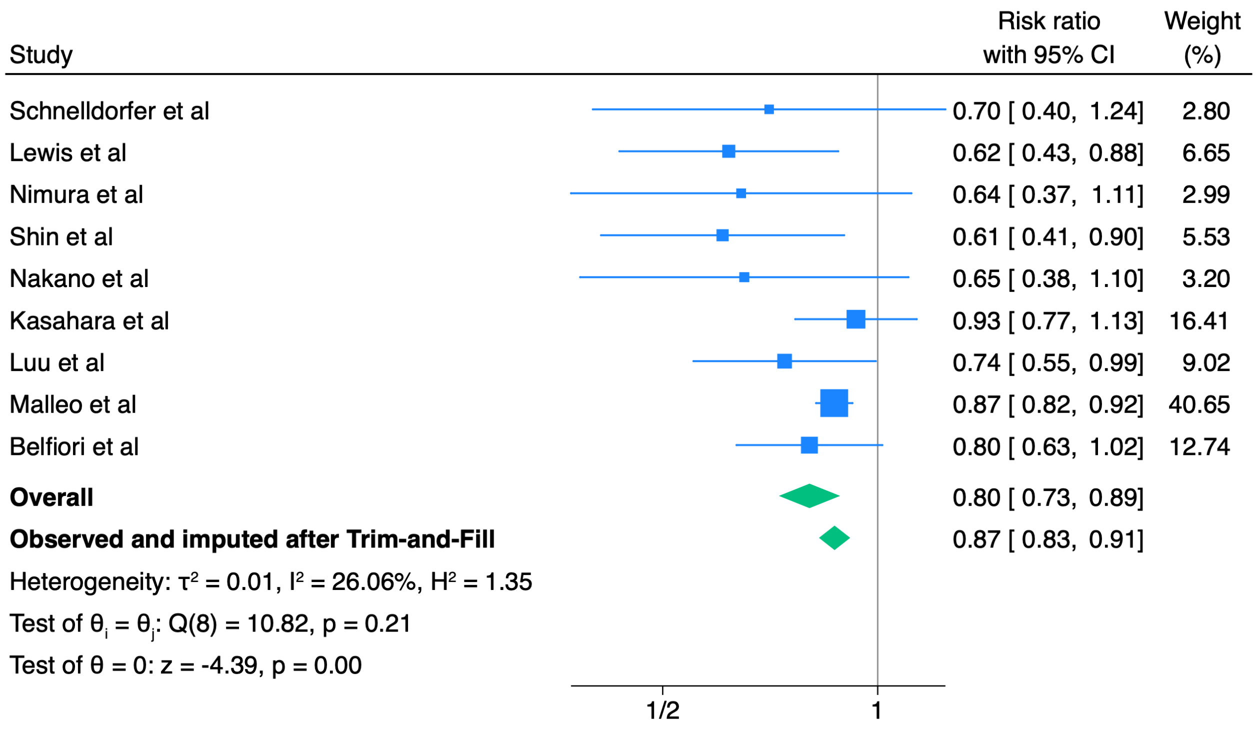

Figure 25. Lymph-Vascular Invasion (LVI), forest plot.

Forest plot of main outcomes, surgical and therpeutic.

Overall Effect favours Long-Term-Survivors if RR > 1 or MD > 0.

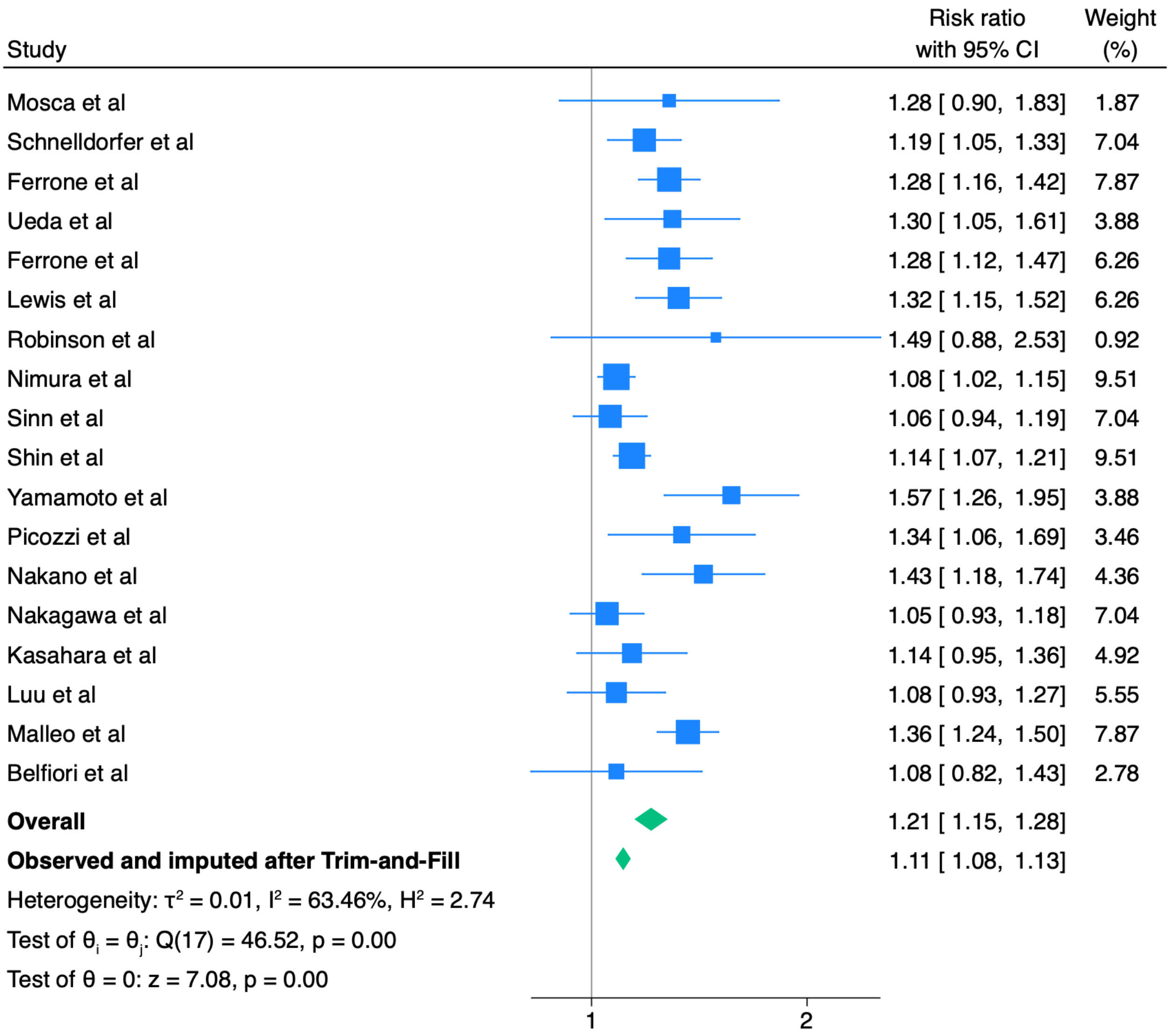

Figure 26. Radical Resection R0, forest plot.

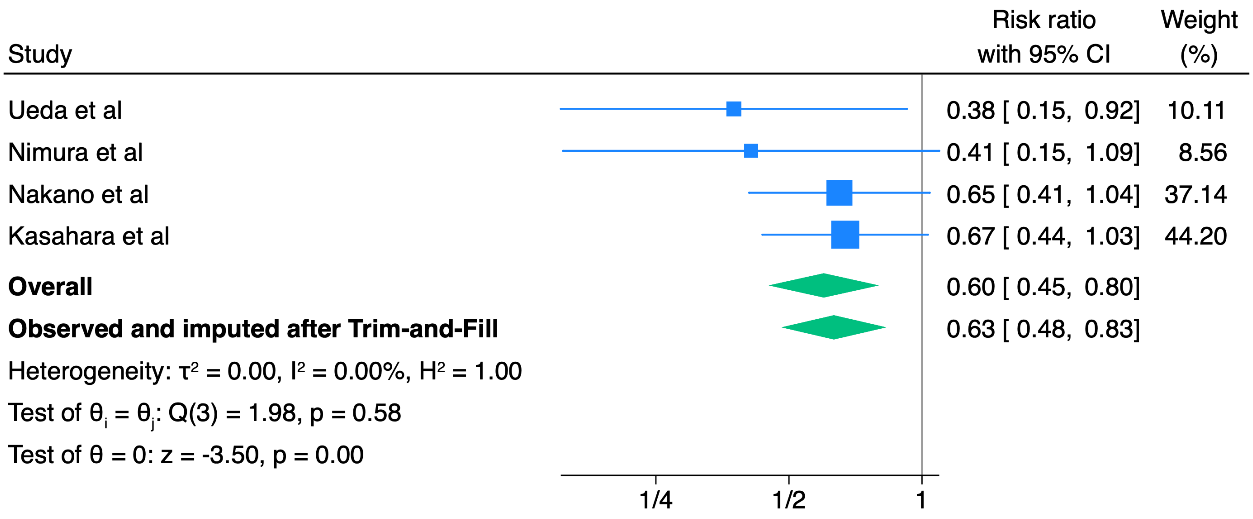

Figure 27. Venous Invasion, forest plot.

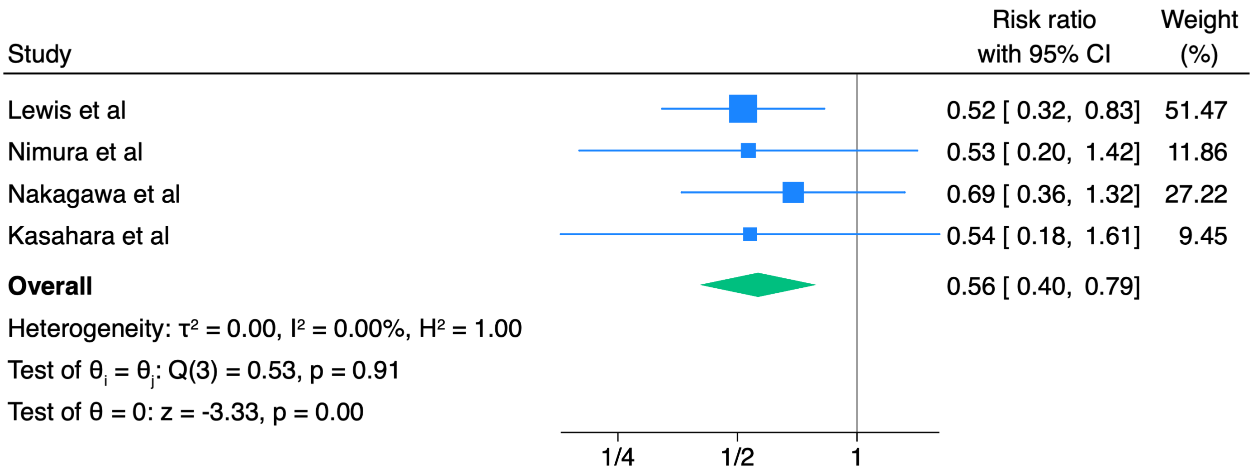

Figure 28. Perioperative Red Blood-Cell Transfusion, forest plot.

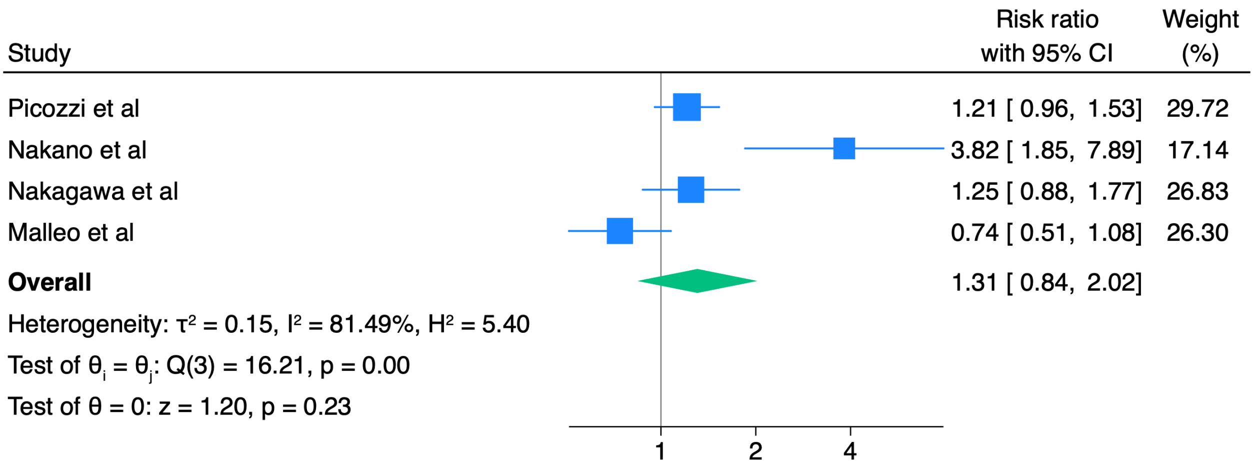

Figure 29. Neoadjuvant chemotherapy, forest plot.

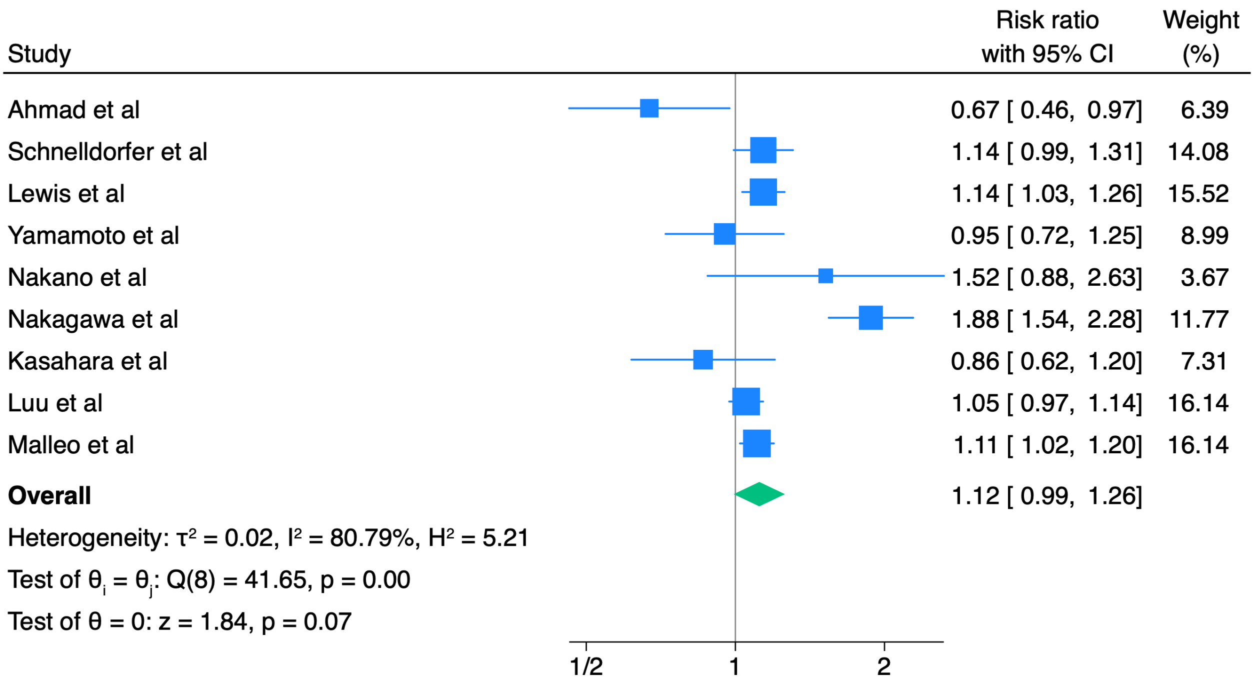

Figure 30. Adjuvant Chemotherapy, forest plot.

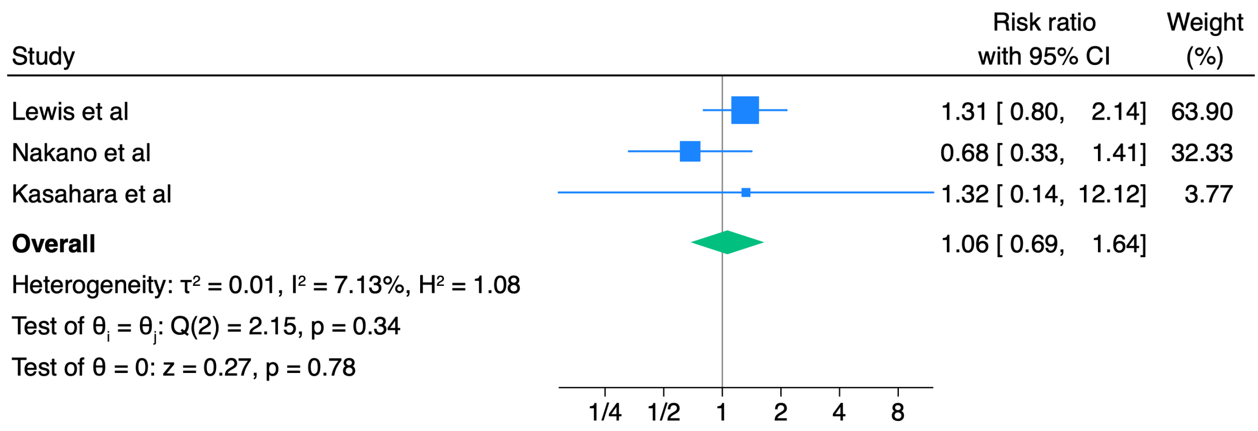

Figure 31. CR-POPF, forest plot.

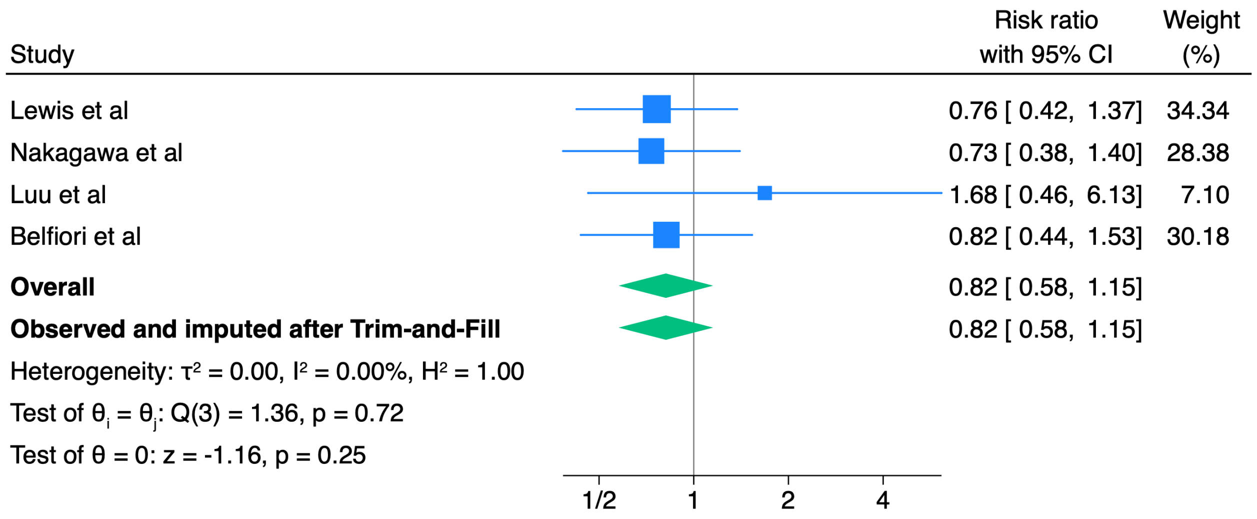

Figure 32. Major Morbidity (Clavien-Dindo ≥ 3), forest plot.

Others forest plots.
Overall Effect favours Long-Term-Survivors if RR > 1 or MD > 0.

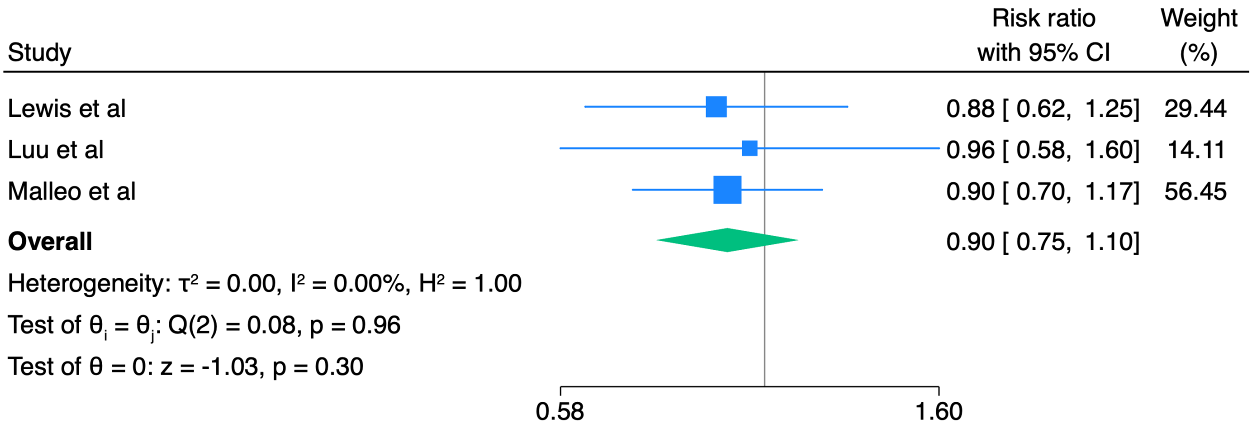

Figure 33. Diabetes, forest plot.

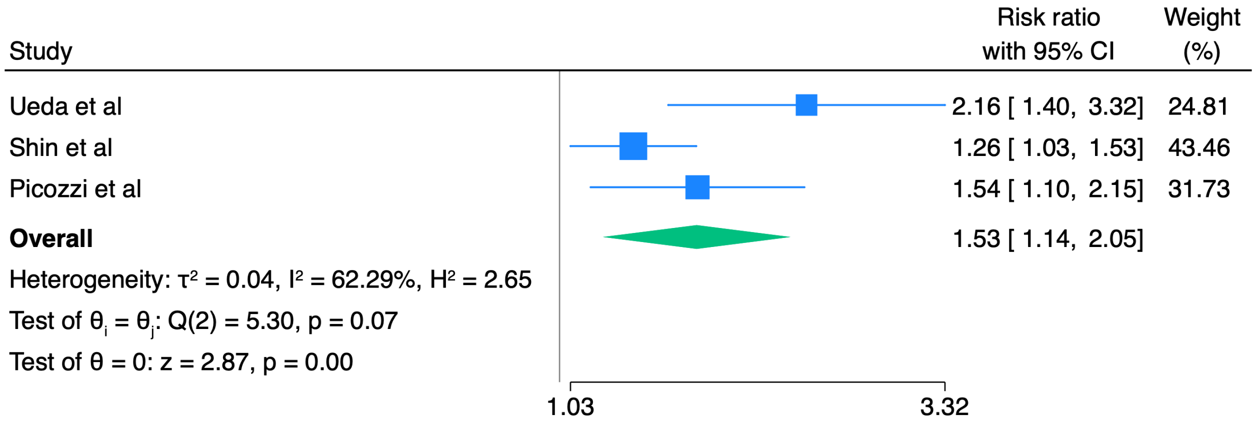

Figure 34. Size < 30 mm, forest plot.

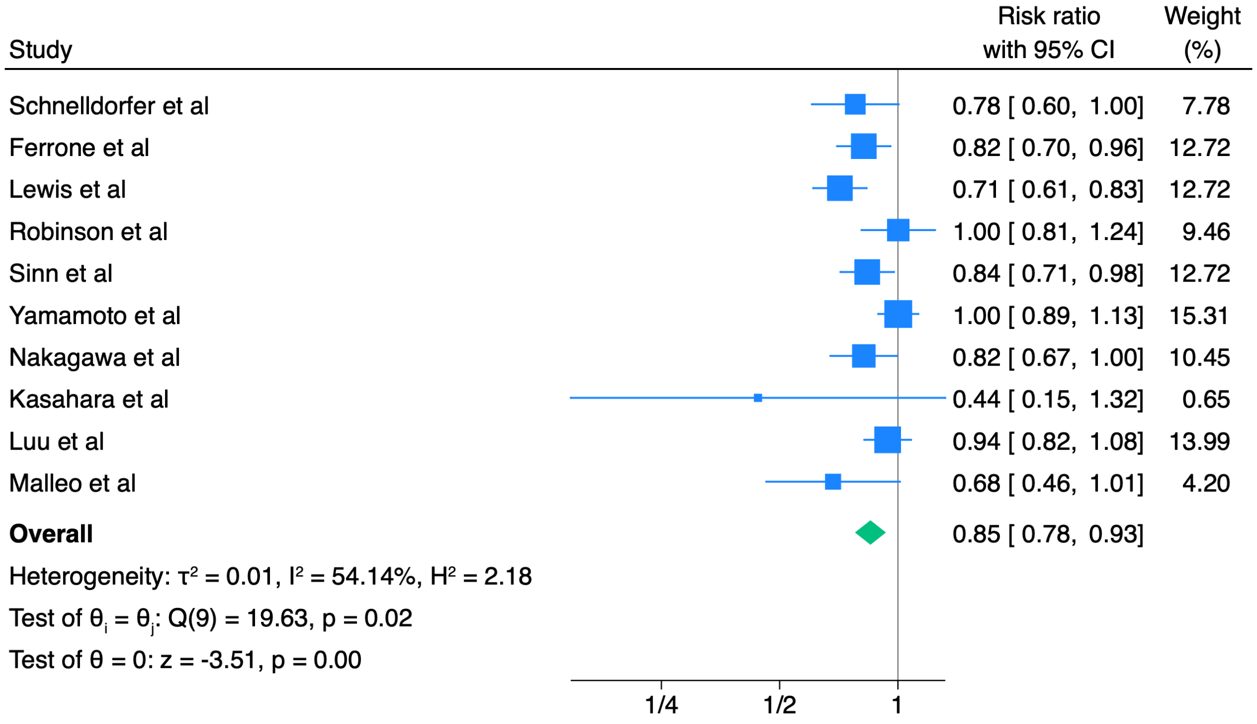

Figure 35. T3-T4, forest plot.

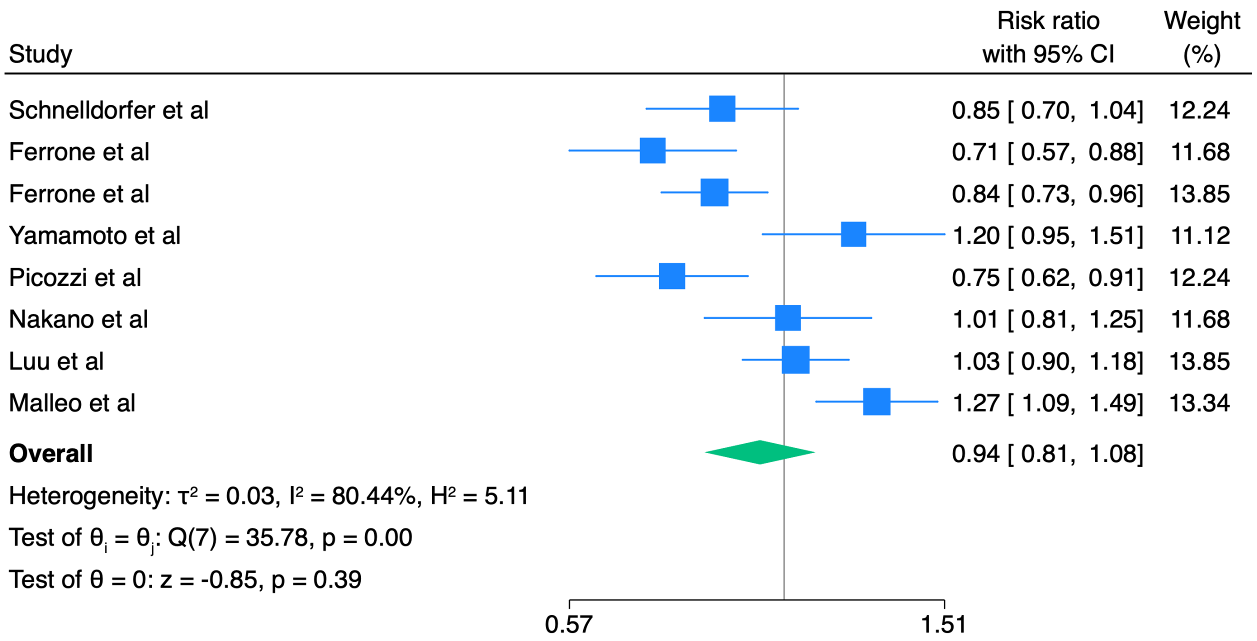

Figure 36. AJCC Stage II, forest plot.

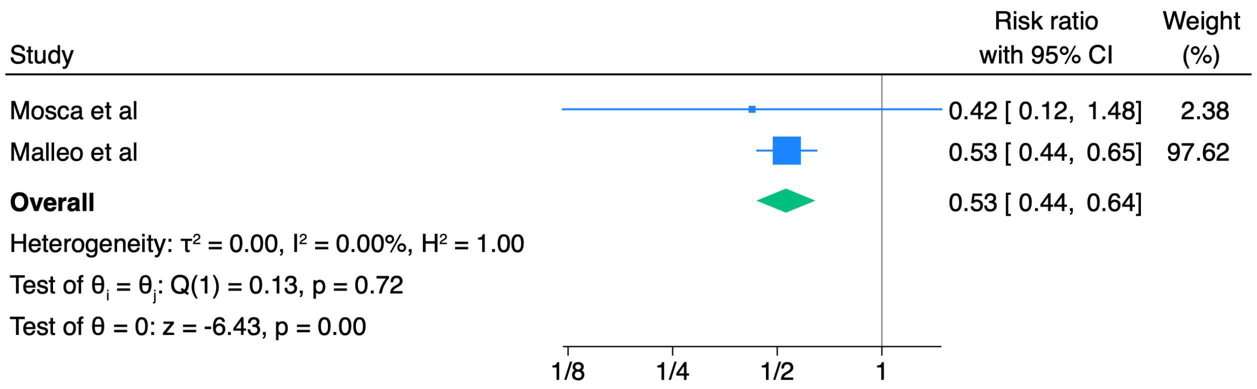

Figure 37. AJCC Stage III, forest plot.

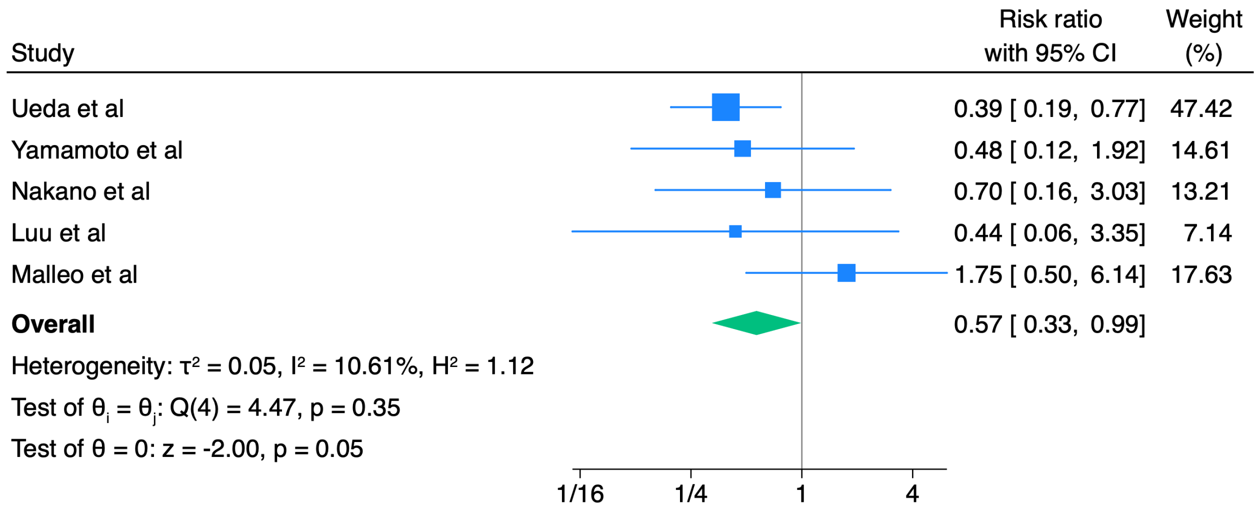

Figure 38. AJCC Stage IV, forest plot.

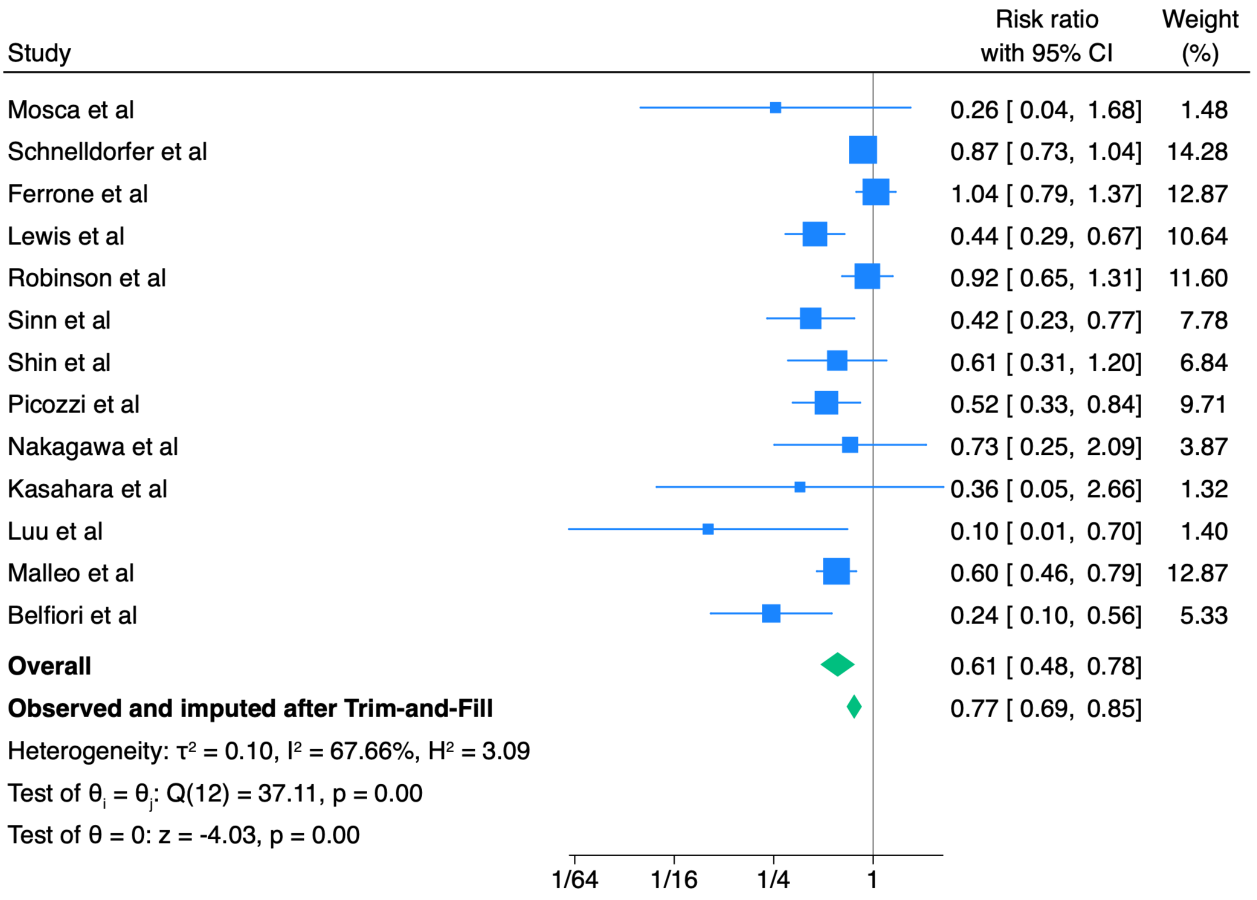

Figure 39. G3, forest plot.

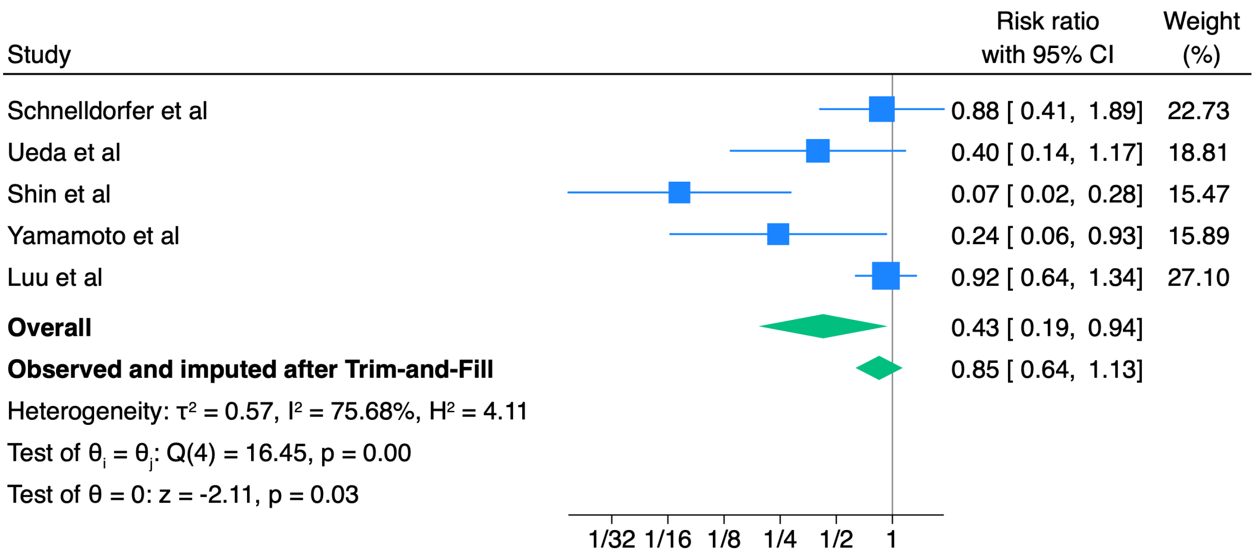

Figure 40. Portal Invasion, forest plot.

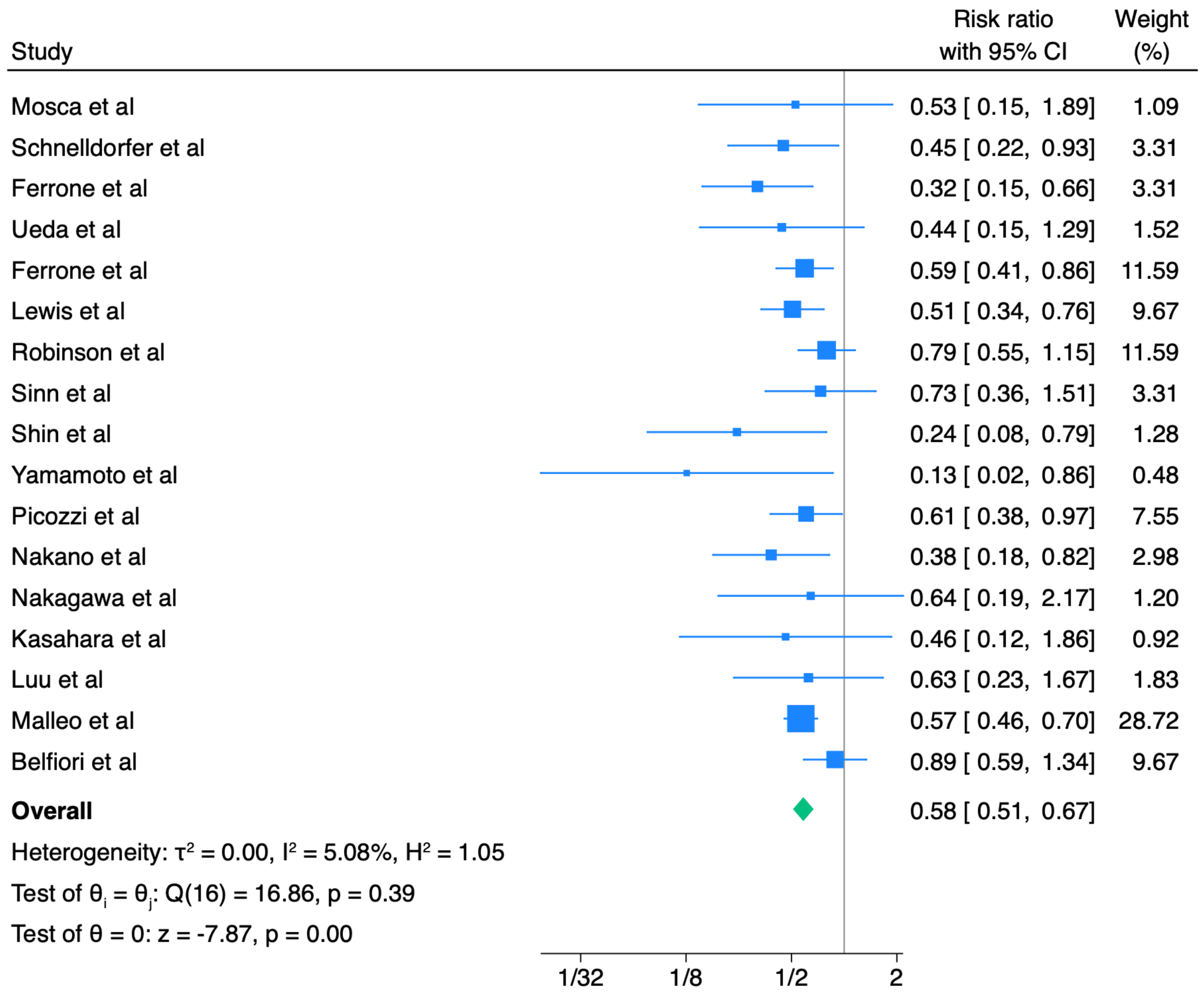

Figure 41. R+, forest plot.

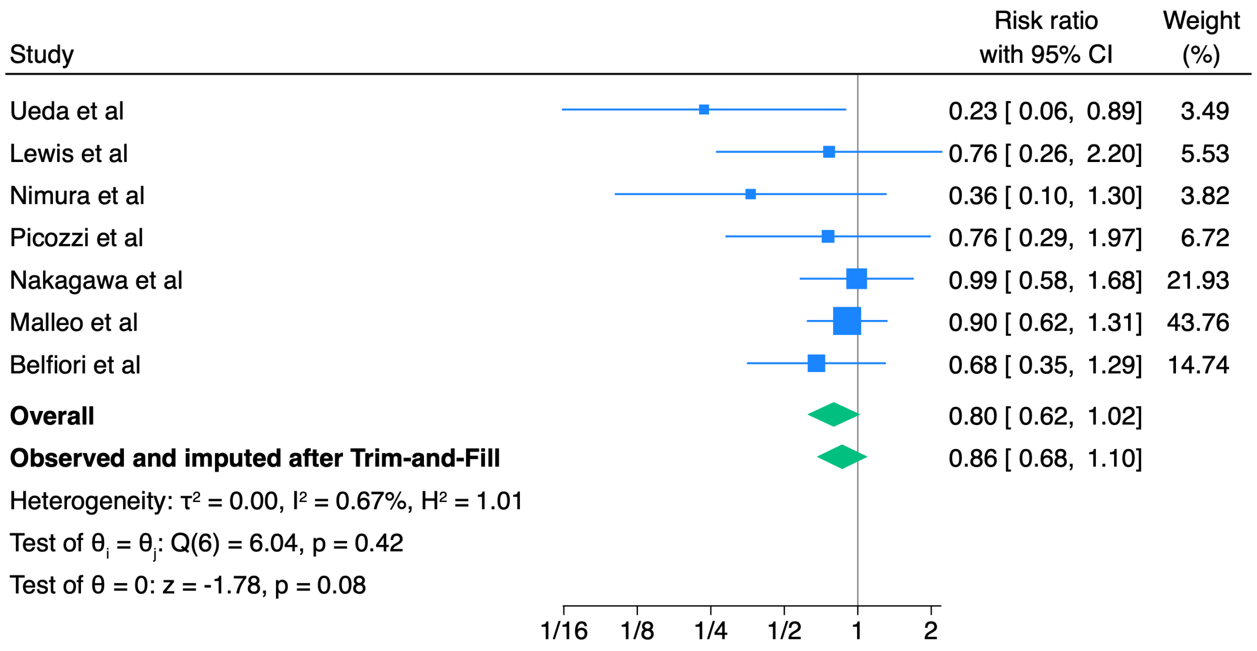

Figure 42. Vascular Resection, forest plot.
