# Supplementary material for: Predictive factors for long-term survival in pancreatic ductal adenocarcinoma that underwent surgery: a systematic review and meta-analysis of literature
Source: Updates Surg. 2025 Sep 1;78(2):509–18. doi: 10.1007/s13304-025-02382-z (PMC13212744; doi:10.1007/s13304-025-02382-z)
Supplement: Supplementary file 2 — Supplementary file2 (PDF 1022 KB) [file 13304_2025_2382_MOESM2_ESM.pdf]

Meta-regression analysis

Supplementary Results.

<u>*Heterogeneity and meta-regression analysis*</u>

Heterogeneity was high for these significative factors: CEA (I^2 99.1%), Ca 19.9 (I^2 98.1%), Size < 30mm (I^2 62.3), T1-2 stage (I^2 81%), T3-4 stage (I^2 54.1%), N0 (I^2 59.4%), grading G1-2 (I2 77.1%), grading G3 (I2 67.7%) and R0 (I2 63.5%). For this reason, a meta-regression analysis was conducted to explain the high heterogeneity, which is shown in Supplementary Material S-2 Table 1 and 2. Regression curves are illustrated in Supplementary Material S-2, Figures 1-4.

The mean age difference between the two groups (β= -223.2 ± 88.3) significantly influenced the mean difference of Ca 19.9 levels between the two groups, but only if 10.000 permutations were carried out (p=0.028). As suggested by R^2, this variation can explain the 64.0% heterogeneity present for this factor. This means that the MD of Ca 19.9 levels increases with the decrease of MD of ages between the two groups. In other words, the more the ages of patients in the STS group increased (MD reduces), the more the differences in Ca 19.9 levels between the two groups reduced.

Another factor that significantly explains the high heterogeneity for MD of Ca 19.9 levels is the RR of adjuvant chemotherapy in two groups (β= -1702.0 ± 271.7; p-value = 0.008), but didn't reach statistical significance after the 10,000 permutations. This variation can explain the 93.7% of heterogeneity. This means that the more the patients undergone to adjuvant chemotherapy in STS group increases (RR decreases), the more the difference of Ca 19.9 between two groups (and heterogeneity) reduces.

Regarding the predictive factor N0, the risk ratio of R0 between two groups significantly influenced the RR of N0 tumors between two groups (β= 1.04 ± 0.47; p value = 0.043), even when 10.000 Monte Carlo permutations were carried out (p=0.025). In particular, the RR of N0 increases with the increase of RR of R0 resection between two groups. As shown in the regression curves, the decrease of R0 resection in LTS group leads to the decrease of N0 tumors in LTS group, and reduces the difference between two groups. This variation can explain the 44.1% of heterogeneity.

Finally, regarding the factor G1-G2, the Eastern/Western localization of studies significantly influenced the RR of G1-G2 tumors between two groups (β= -0.22 ± 0.11), but only when 10.000 Monte Carlo permutations were carried out (p=0.044). This variation can explain the 35.0% of heterogeneity. In other words, the more the studies were conducted in Eastern Countries, the lower the difference between two groups (RR of G1-G2 reduces).

For others high-heterogeneous predictive factors, covariates did not significantly explain the high heterogeneity even when the 10,000 permutations were carried out.

Covariates	CEA (p-val)	CA19.9 (p-val)	T1-2 (p-val)	T3-4 (p-val)	Size<30 (p-val)	N0 (p-val)	R0 (p-val)	G1-G2 (p-val)	G3 (p-val)
Year	0.885	0.679	0.756	0.660	0.535	0.123	0.707	0.305	0.209
East/West	0.626	0.232	0.997	0.413	0.953	0.287	0.512	***0.067***	0.968
MINORS	0.348	0.449	0.187	0.487	0.289	0.825	0.133	0.886	0.381
Age (MD)	0.481	***0.086***	0.158	0.326	-	0.211	0.159	0.335	0.691
Male sex (RR)	0.153	0.444	0.155	0.356	0.269	0.215	0.889	0.380	0.187
ASA I-II (RR)	-	0.890	0.512	0.130	-	0.885	0.407	0.614	0.555
BMI (MD)	-	0.629	-	-	-	0.502	0.515	-	-
T1-2 (RR)	-	***0.095***	-	-	-	0.205	0.137	0.881	0.415
N0 (RR)	0.720	0.720	0.547	***0.088***	-	-	***0.076***	0.873	0.332
R0 (RR)	0.799	0.570	0.328	0.333	0.510	***0.043***	-	0.308	0.183
G1-2 (RR)	-	0.378	0.779	0.964	-	0.458	0.529	-	-
Neo CT (RR)	-	0.686	-	-	-	0.396	0.625	0.998	0.809
Adj CT (RR)	0.154	***0.008***	0.276	0.719	-	0.856	0.452	0.419	0.643
CEA (MD)	-	0.139	-	-	-	0.661	0.761	-	-
Ca 19.9 (MD)	***0.080***	-	0.155	0.402	-	0.660	0.540	0.360	0.713

Table 1. Meta regression analysis for high heterogeneous and significative predictive factors.
Legend: MD = Mean Difference; RR = Risk Ratio; BMI = Body Mass Index; T1-2 = T parameter in TNM staging; N0 = N parameter in TNM staging; R0 = radical resection with negative margins; G1-2 = pathological grading; Neo CT = Neoadjuvant chemotherapy; Adj CT = Adjuvant chemotherapy; p-val = p-value.

Covariates by outcome	Number of studies	Beta coefficient ± SE	Adjusted R^2	P-value	P-value ± SE after Monte Carlo Permutation
**CEA**					
Ca 19.9	3	0.01 (<0.01)	98.0	0.080	0.341 (0.005)
**CA 19.9**					
Age (MD)	5	-223.2 (88.3)	64.0	0.086	***0.028 (0.002)***
T1-2 (RR)	4	-806.8 (268.5)	75.1	0.095	0.127 (0.003)
Adj CT (RR)	5	-1702.0 (271.7)	93.7	***0.008***	0.059 (0.002)
**T3-4**					
N0 (RR)	9	0.06 (0.03)	47.5	0.088	0.092 (0.003)
**N0**					
R0 (RR)	15	1.04 (0.47)	44.1	***0.043***	***0.025 (0.002)***
**R0**					
N0 (RR)	15	0.09 (0.05)	16.8	0.076	0.074 (0.003)
**G1-G2**					
East-West	13	-0.22 (0.11)	35.0	0.067	***0.044 (0.002)***

Table 2. Meta regression analysis for high heterogeneous and significative predictive factors.
Legend: MD = Mean Difference; RR = Risk Ratio; T3-4 = T parameter in TNM staging; N0 = N parameter in TNM staging; R0 = radical resection with negative margins; G1-2 = pathological grading; Adj CT = Adjuvant chemotherapy. SE standard error; R^2 = relative reduction in between-study variance, the value indicates the proportion of between-study variance explained by covariate.

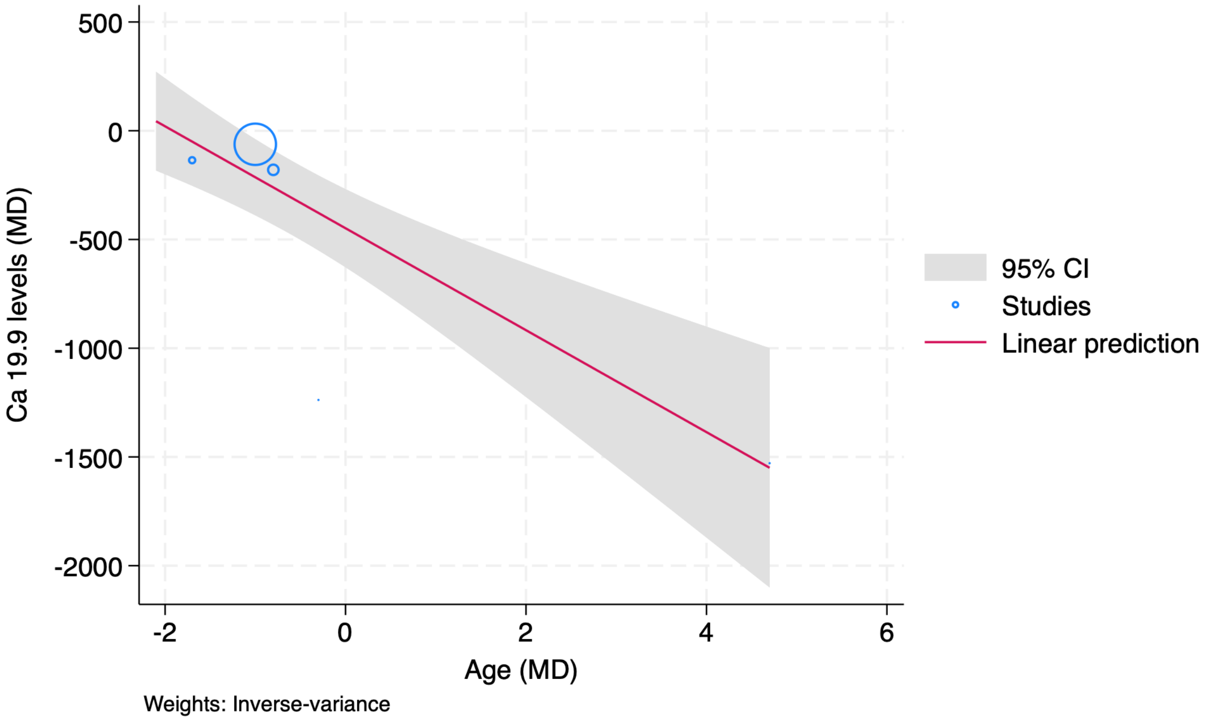

Figure 1. Bubble plot with a fitted meta-regression line describing the relationship between the Mean Difference (MD) of age and the Mean Difference (MD) of Ca 19.9.

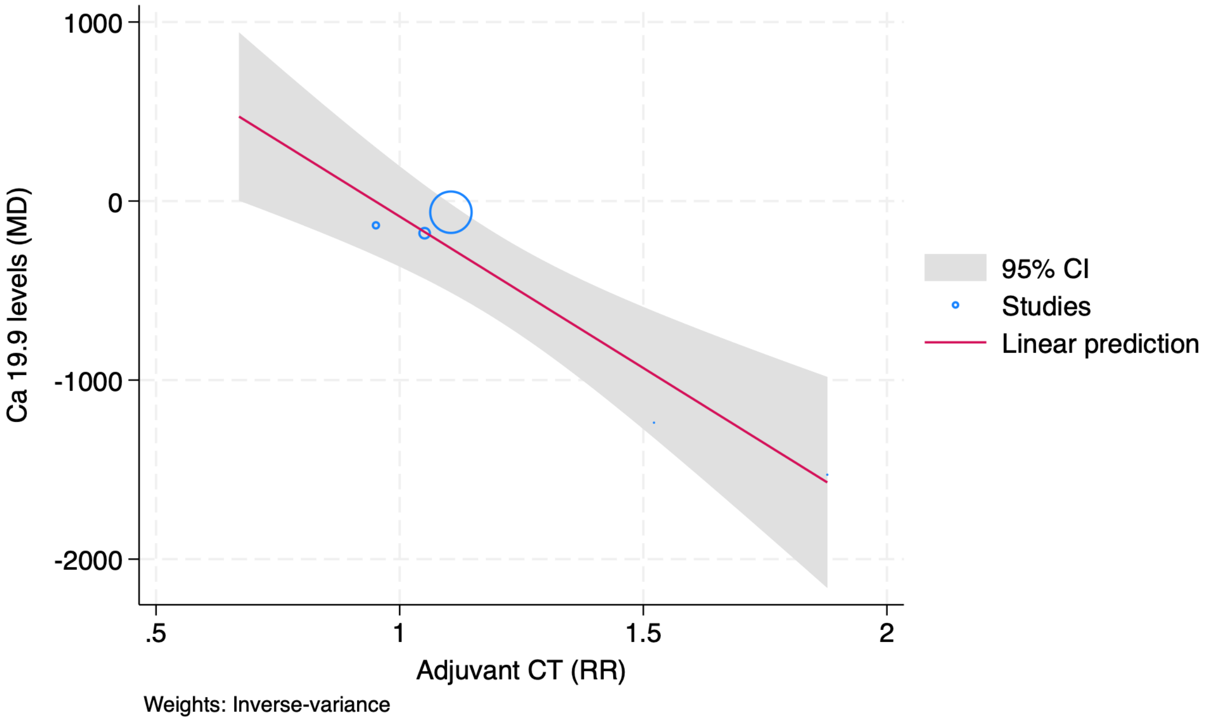

Figure 2. Bubble plot with a fitted meta-regression line describing the relationship between the Risk Ratio (RR) of adjuvant chemotherapy and the Mean Difference (MD) of Ca 19.9.

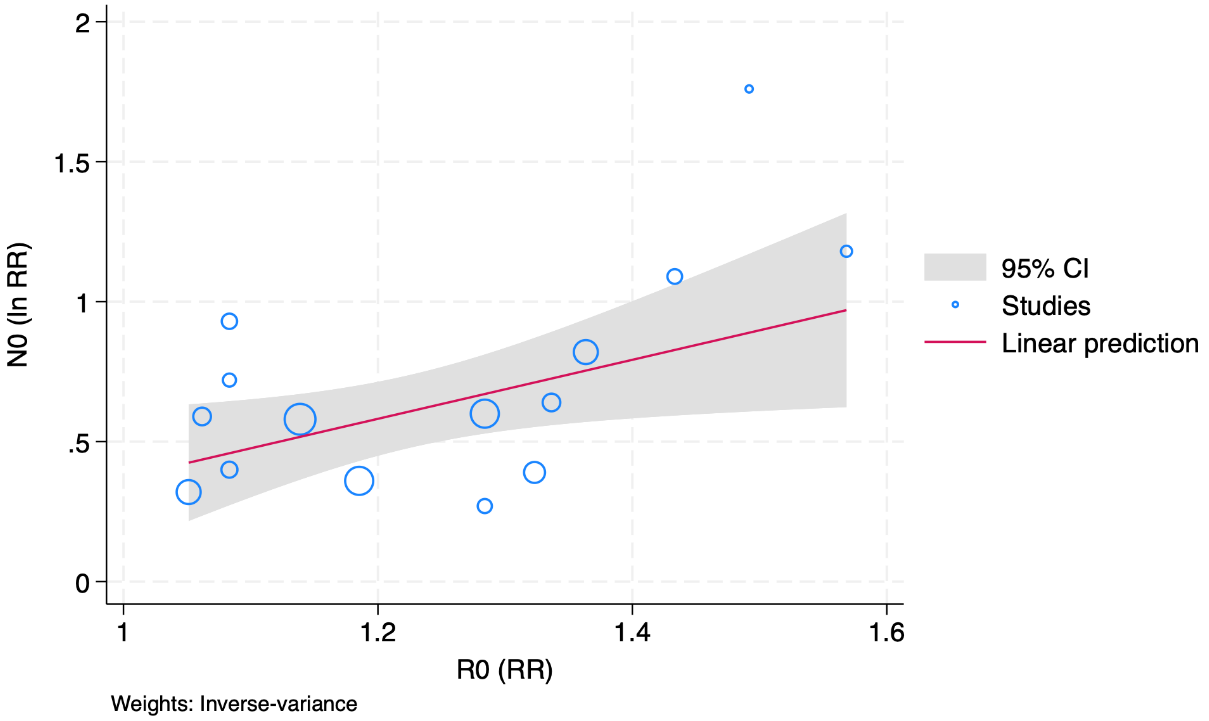

Figure 3. Bubble plot with a fitted meta-regression line describing the relationship between the Risk Ratio (RR) of R0 resections and the Risk Ratio (ln RR) of N0 tumors.

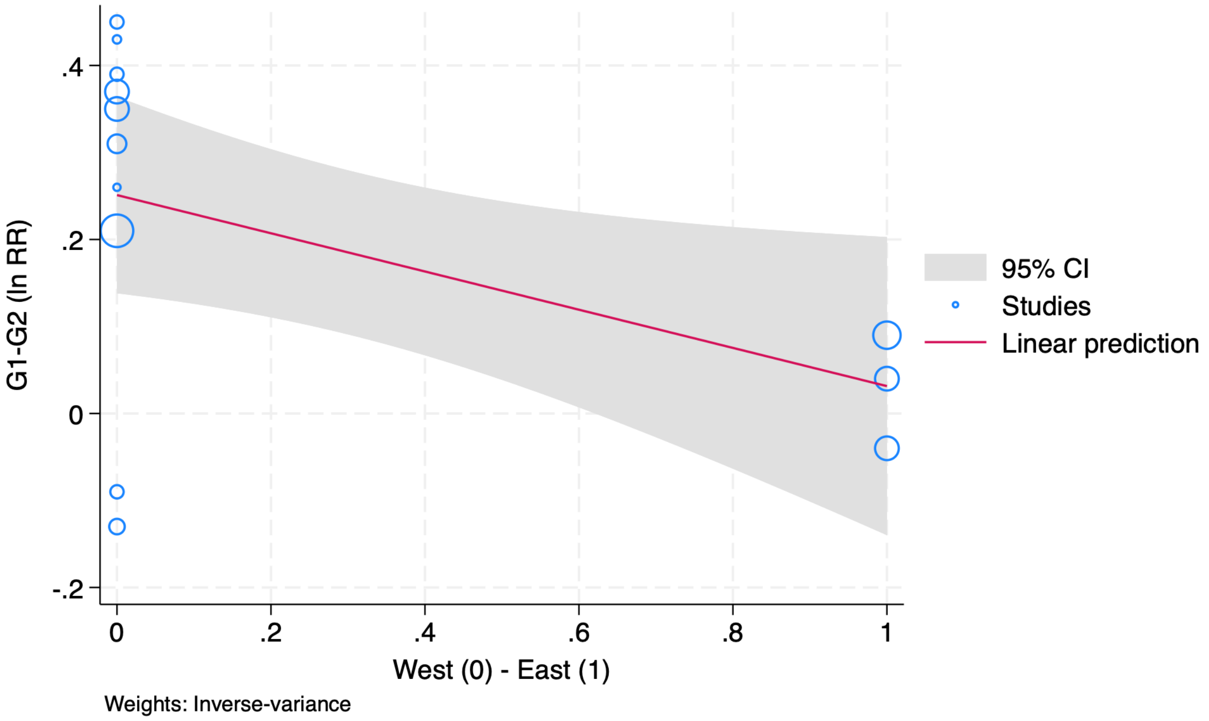

Figure 4. Bubble plot with a fitted meta-regression line describing the relationship between Western or Eastern studies and the Risk Ratio (ln RR) of low-grade tumors (G1-G2).

Neoadjuvant and adjuvant chemotherapy role

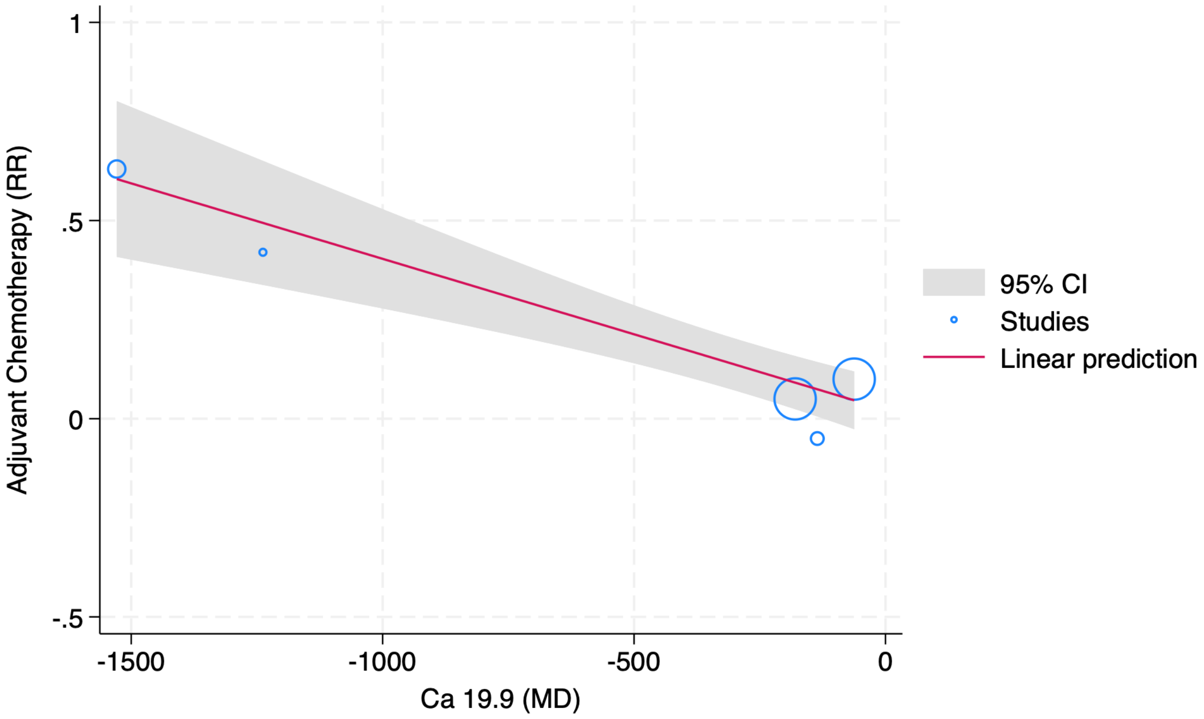

Figure 5. Bubble plot with a fitted meta-regression line describing the relationship between Ca 19.9 levels and the Risk Ratio of Adjuvant Chemotherapy between two groups.

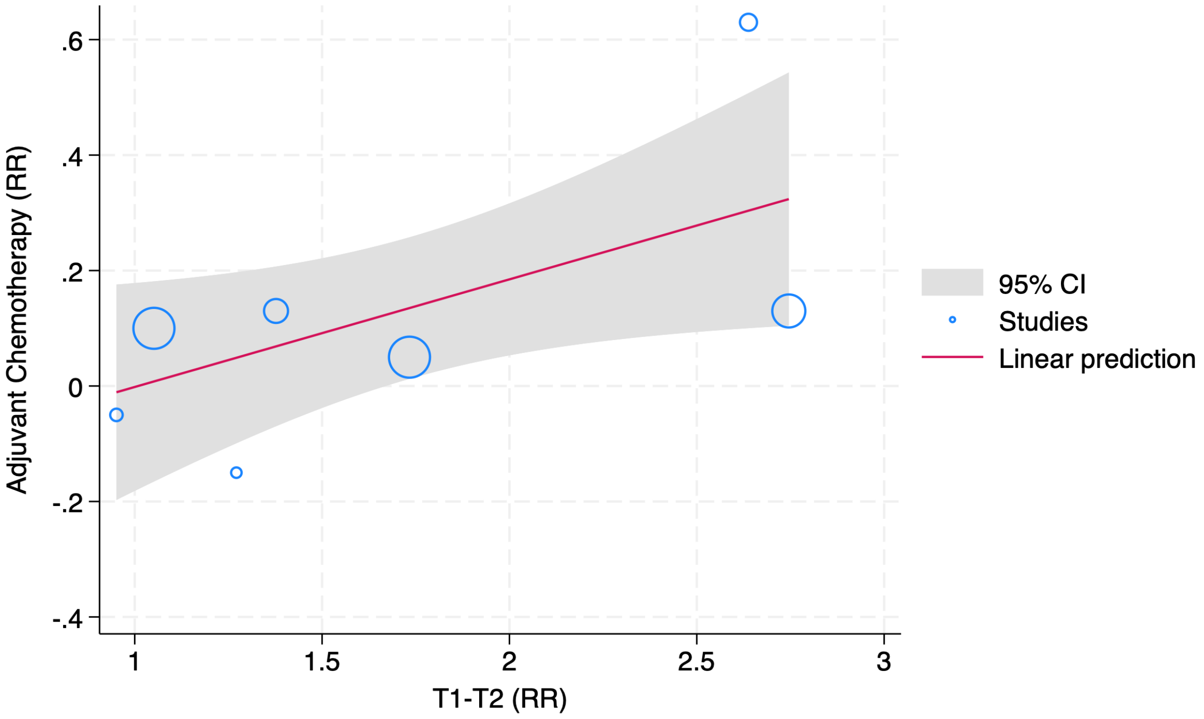

Figure 6. Bubble plot with a fitted meta-regression line describing the relationship between the Risk Ratio of T1-T2 tumors between two groups and the Risk Ratio of Adjuvant Chemotherapy between two groups.

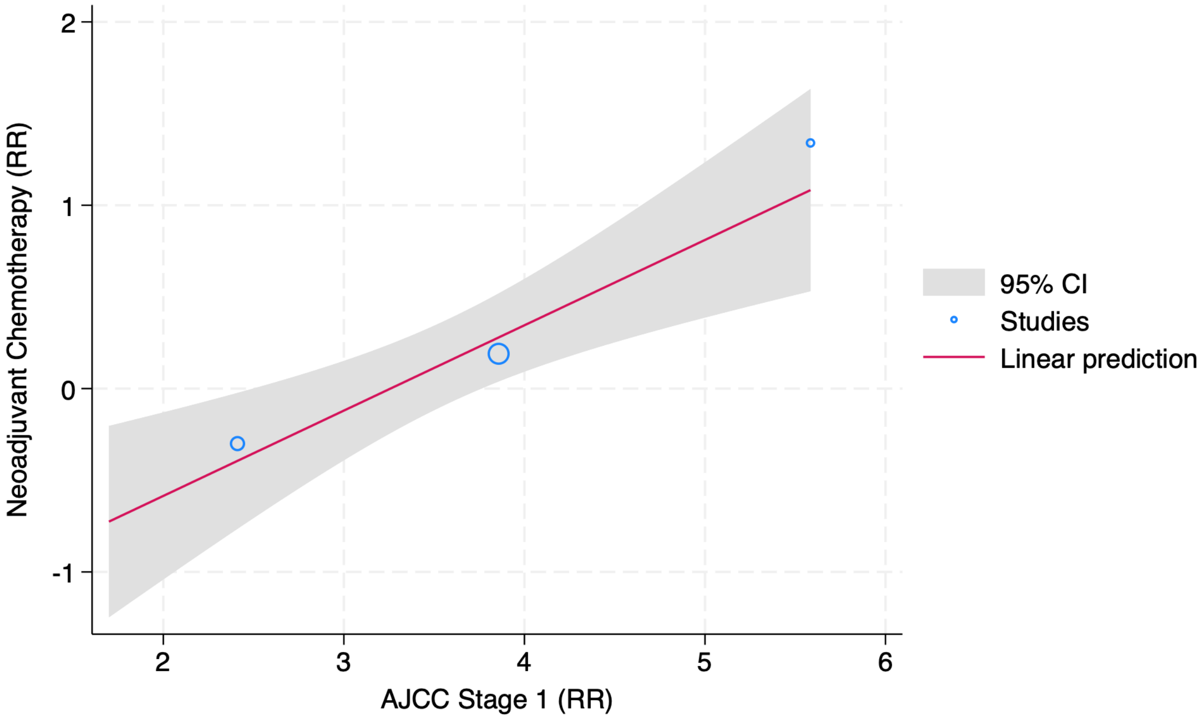

Figure 7. Bubble plot with a fitted meta-regression line describing the relationship between the Risk Ratio of AJCC Stage 1 tumors between two groups and the Risk Ratio of Neodjuvant Chemotherapy between two groups.

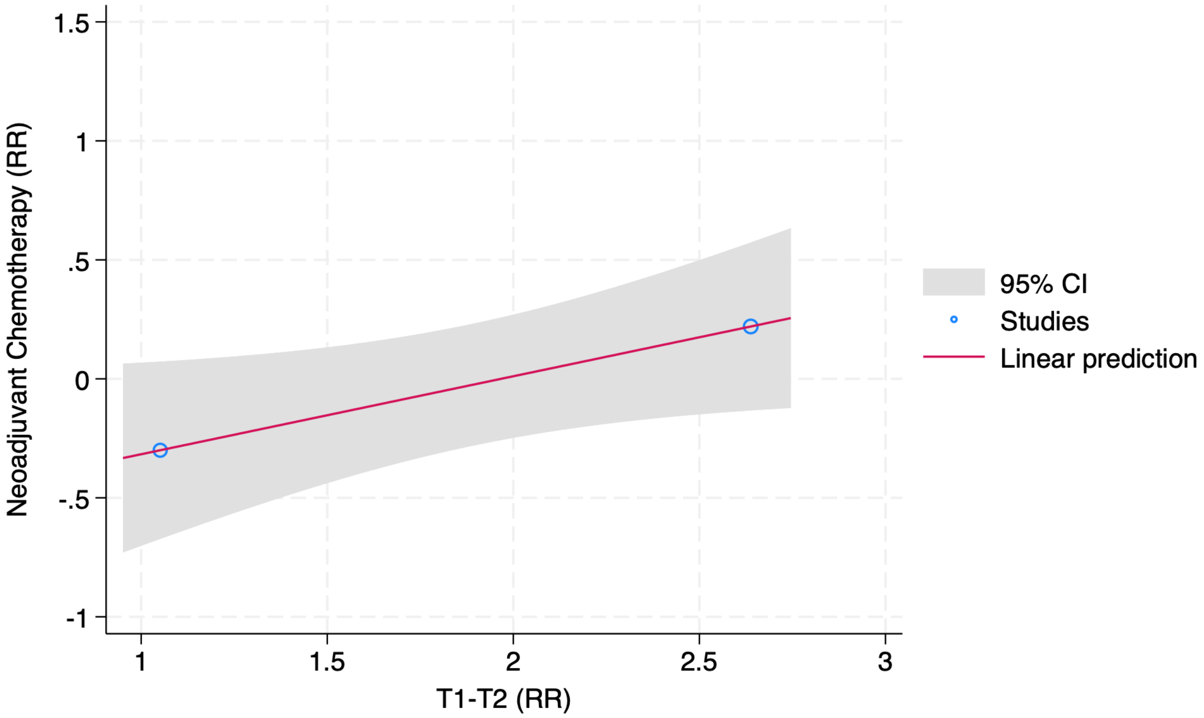

Figure 8. Bubble plot with a fitted meta-regression line describing the relationship between the Risk Ratio of T1-T2 tumors between two groups and the Risk Ratio of Neoadjuvant Chemotherapy between two groups.
